# Comprehensive treatment of microvascular angina in overweight women – a randomized controlled pilot trial

**DOI:** 10.1371/journal.pone.0240722

**Published:** 2020-11-05

**Authors:** Kira Bang Bove, Malin Nilsson, Lene Rørholm Pedersen, Nicolai Mikkelsen, Hannah Elena Suhrs, Arne Astrup, Eva Prescott

**Affiliations:** 1 Department of Cardiology, Bispebjerg-Frederiksberg Hospital, University of Copenhagen, Copenhagen, Denmark; 2 Department of Endocrinology, Bispebjerg-Frederiksberg Hospital, University of Copenhagen, Copenhagen, Denmark; 3 Department of Nutrition, Exercise and Sports (NEXS), Faculty of Science, University of Copenhagen, Copenhagen, Denmark; Kurume University School of Medicine, JAPAN

## Abstract

**Aims:**

Coronary microvascular dysfunction (CMD) carries a poor cardiovascular prognosis and may explain angina in women without obstructive coronary artery disease (CAD). Currently, no evidence-based treatment for CMD exists. We investigated whether reducing cardiovascular risk factors improves symptoms and microvascular function in women with non-endothelial dependent CMD and no obstructive CAD.

**Methods:**

We randomized 62 women aged 40–75, with body mass index (BMI) >25 kg/m^2^, angina ≥monthly, and coronary flow velocity reserve (CFVR) ≤2.5 to a 24-week intervention comprising low energy diet, exercise training, and optimized treatment of hypertension, dyslipidemia and diabetes or to control. Patients were assessed before randomization and after 24 weeks. Primary outcomes were CFVR assessed by transthoracic Doppler stress-echocardiography and angina burden by Seattle Angina Questionnaire (SAQ). Secondary outcomes were exercise capacity, body composition, glycemic control, myocardial function, and anxiety and depression symptoms.

**Results:**

Fifty-six participants (90%) completed the study. Median (IQR) age was 65.2 (57.1;70.7) years, BMI was 30.1 (28.4;32.7) kg/m^2^. The intervention resulted in relevant improvement in angina symptoms (9-21-point increase on SAQ-scales (all p<0.01)) but had no effect on CFVR (p = 0.468). Mean (CI) weight loss was 9.6 (7.80;11.48) kg, (p<0.0001). There was a significant mean (CI) decrease in depression symptoms = 1.16 (0.22;2.12), triglycerides = 0.52 (0.25;0.78) mmol/L, total cholesterol = 0.55 (0.12;0.98) mmol/L, and HbA1c in diabetics = 27.1 (1.60;52.6) mmol/mol but no effect on other secondary outcomes.

**Conclusion:**

A major weight loss and intensified risk factor control resulted in significantly improved angina burden but no improvement of coronary microvascular function among women with microvascular angina.

## Introduction

Most women with suspected angina pectoris have no obstructive coronary artery disease (CAD) [1] yet many continue to have symptoms resulting in frequent hospital re-admissions, anxiety and depression, impaired quality of life, and an adverse prognosis [2]. A large proportion of these patients have coronary microvascular dysfunction (CMD) and angina due to CMD, termed microvascular angina (MVA). There is strong evidence that CMD predicts further cardiovascular events [1, 3–5] and shares common cardiovascular risk factors with obstructive CAD [6, 7]. A few intervention studies have indicated improved coronary microvascular function after treatment of modifiable risk factors including exercise training, weight loss [8, 9] and optimized medical treatment [10, 11].

To our knowledge, no randomized controlled trial has examined whether a comprehensive intervention targeting CMD risk factors improves MVA. We hypothesized that amelioration of risk factors for CMD would improve both angina and coronary microvascular function. We tested this hypothesis by randomizing symptomatic women with CMD and no obstructive epicardial disease to an intervention comprising weight loss, aerobic interval training (AIT), and optimal medical treatment of hypertension, dyslipidemia and diabetes versus usual care.

Abnormalities in endothelial and non-endothelial pathways of coronary macro- and microvascular function can be assessed by intracoronary infusion of vasoactive substances [12, 13]. However, non-invasive methods may be favorable from ethical and economic considerations. In this study, we assessed coronary microvascular function non-invasively by transthoracic Doppler echocardiography (TTDE). The method is free from radiation, is highly feasible, reproducible and correlate well with invasive methods [14–18]. However, CMD caused by endothelial dysfunction may not be detected by this method. Non-invasive alternatives for assessing endothelial dependent coronary microvascular function count flow mediated dilation and digital reactive hyperemia index. However, these methods have shown not to correlate with CFVR by TTDE in previous studies on our population and are therefore not evaluated in this study [19, 20].

## Methods

### Study design and population

We included 62 women from December 2016 to April 2018. Participants were randomized 1:1 to either intervention or control (usual care). On the first examination day, each participant was assigned a number between 1–100 that had not already been allocated to another participant. If the participant was still eligible for inclusion after baseline examinations had been conducted, the participant’s number was randomly allocated to either intervention or control. The randomization was performed by a researcher at Bispebjerg Hospital, who was impartial and not related to the study group. Also, the randomization program was kept secret by this third person. STATA II, ralloc program was used, permuting random block sizes of 2, 4 and 6.

All participants were recruited from the iPOWER (ImProve diagnOsis and treatment of Women with angina pEctoris and micRovessel disease) cohort [21] comprising 1856 women, consecutively included, with symptoms of ischaemia but no obstructive lesions on invasive angiography (<50% epicardial stenosis). Inclusion criteria were CMD defined as a coronary flow velocity reserve (CFVR) ≤2.5, age 40–75, angina symptoms more than once monthly and overweight (body mass index (BMI) >26 kg/m^2^; or BMI 25–26 and waist-hip-ratio >0.8).

Patients were excluded in case of severe renal failure (eGFR <30), hepatic comorbidity, chronic alcohol abuse, allergies to content of the low energy diet, atrial flutter or fibrillation at the time of stress-echocardiography, physical or mental disabilities contraindicating or hampering diet or exercise training.

**Fig 1:**
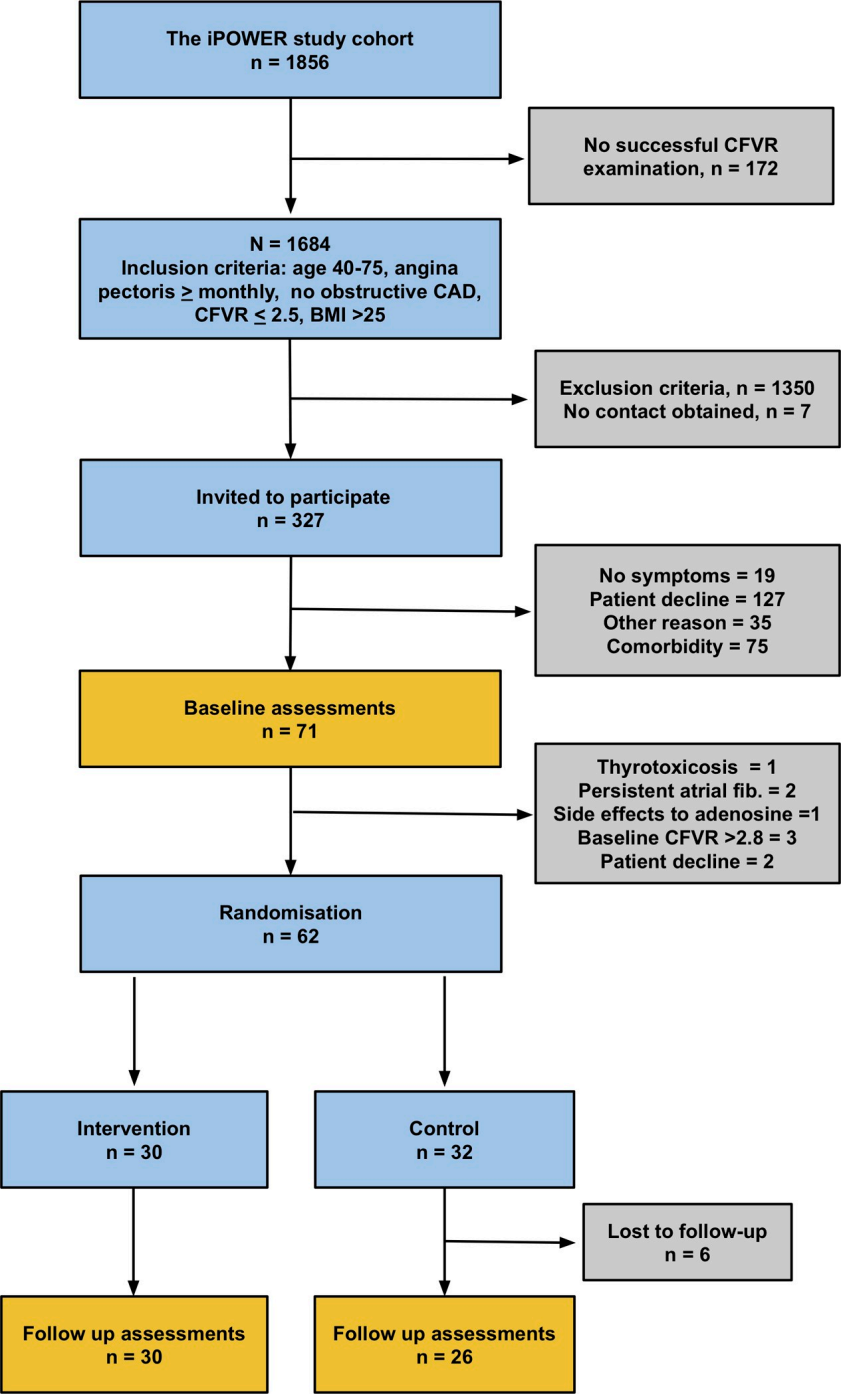
Study flowchart. Inclusion and exclusion flow chart of the two study groups.

For patients accepting participation, CFVR was re-assessed. Accepting a small variation in CFVR, patients with CFVR >2.8 at this second assessment were excluded. **Fig 1**is an inclusion flow chart of the study.

### The intervention

The intervention was multidisciplinary, targeting cardiovascular risk factors through weight loss, exercise training and optimizing blood pressure, lipids and glycemic control. We aimed at a body weight loss of at least 10% without significant loss of muscle mass, achieved through low energy diet (LED) combined with exercise training (AIT and resistance exercise) [22] and risk factor control through medication following the European Guidelines [23]. The intervention was carried out at the Department of Cardiology and Rehabilitation at Bispebjerg Frederiksberg University Hospital.

Components and goals of the intervention is outline in **S1 Table**, participant’s study plan in **S1 Fig** and medication flow chart in **S2 Fig**.

#### Low energy diet

Weight loss was achieved by 12 weeks of LED delivered by the Cambridge Weight Plan, Northants, UK. The LED contained 800–1200 kcal/day and covered the daily need for macro- and micronutrients, essential fatty acids and recommended daily doses of vitamins and minerals [24]. The LED was followed by 12 weeks of maintenance diet high in protein and with a low glycemic index modified from the DiOGenes study [25]. Taking into consideration that the participants had CMD the high protein diet was adapted to resemble the Mediterranean diet, low in saturated fat (<10%) and protein sources mainly from fish, poultry, egg, dairy, and vegetables as recommended to heart patients [26]. Throughout the intervention, participants were closely supervised by dieticians through 2 individual meetings and 6 group sessions during the LED, followed by 3 group sessions during the weight maintenance period.

#### Exercise training

The exercise program consisted of 45–60 minutes group sessions twice weekly throughout the 24-week intervention period. All training sessions were supervised by a physiotherapist. To adapt to the concomitant intensive weight loss program the training was initially modest in load and intensity primarily consisting of short AIT sessions and resistance exercise of the large muscle groups. Home-based training was accepted if participants were unable to attend all the sessions and compliance was self-reported. We defined lack of training compliance as <50% attendance to training sessions or home training.

### Control group–usual care

The control group were followed by their general practitioner as usual. In case of untreated hypertension, dyslipidemia or diabetes mellitus the participants were advised to contact their general practitioner for treatment according to European Guideline targets.

### Primary outcomes

#### Coronary microvascular function

Microvascular function was determined by transthoracic Doppler stress echocardiography (TTDE) measuring the CFVR of the left anterior descending artery (LAD) before and during a 6-minute infusion (0.14 mg/kg/min) with adenosine. CFVR was calculated as the ratio of the flow velocity at maximal hyperaemia and the flow velocity at rest. A CFVR ≤2.5 was definitory for CMD in this study [27]. A 2.7–8 MHz transducer (GE Vivid 6S probe) was used for the CFVR assessment. The examinations were double analyzed after end of study by another blinded expert. The first reading was used unless estimates differed more than 0.2, in which case the experts re-analyzed the examination and reached agreement.

Prior to the CFVR examination, participants were instructed to abstain from food and drinks containing significant amounts of caffeine or methylxanthine (coffee, tea, chocolate, cola and banana) for 24 hours and be abstinent from tobacco for 8 hours (overnight). Anti-ischemic and anti-hypertensive medication including diuretics were also paused for 24 hours, and medication containing dipyridamole was paused for 48 hours.

#### Angina burden

Angina burden was determined by the validated, self-administered Seattle Angina Questionnaire (SAQ) [28, 29]. SAQ evaluates five dimensions of functional status: anginal stability, angina frequency, physical limitation, treatment satisfaction and quality of life. A score between 0–100 range was calculated for each scale. Higher scores indicate less symptom burden. Because each scale monitors a unique dimension of CAD, no summary score is generated. A score of less than 100 on the angina frequency scale indicates angina at least once a month. Physical functioning and quality of life were defined by the SAQ physical limitation and quality of life scales [28]. A score change of 10 points is considered to be perceptible to patients and therefore clinically relevant, while a change of 20 points is considered substantial [28].

### Secondary outcomes

Participants filled out both questionnaires, SAQ and HADS, at home a few days prior to the baseline or follow-up appointment in the clinic, or alone in a sitting room on the day of arrival.

#### Body composition

We aimed at a 10% weight loss with minimal loss of muscle mass. To estimate body weight, body fat mass and fat free mass (FFM) we performed a whole-body dual X-ray absorptiometry scan (GE Lunar iDXA Forma Version 15, GE Lunar Corp, Madison, WI, USA) X-ray absorptiometry (DEXA). Waist circumference was measured halfway between the lower rib and the iliac crest and hip circumference at the maximal gluteal protuberance. Body weight was also measured on a standard scale after minimum 6 hours fast, using the same scale at baseline and follow-up.

#### Cardiorespiratory fitness

A cardiopulmonary exercise test was performed using a bicycle ergometer (Corival, Lode, Germany) with breath-by-breath gas exchange measurements (Jaeger, Vyntus CPX, Germany). Participants were encouraged to continue cycling until exhaustion. Levelling off of VO_2_ despite increasing workload was used as a parameter of a valid test. We aimed at a peak respiratory exchange ratio (RER) >1.10. VO_2_peak and peak RER were determined at peak effort with 15 second average measurements of VO_2_ [30]. VO_2_peak is expressed as: VO_2_peak_total_ (mL/min), VO_2_peak_bw_ (mL/kg body weight/min) and VO_2_peak_FFM_ (mLO_2_/kg FFM^0.67^/min) using FFM derived from the DEXA scan. Predicted VO_2_peak was calculated using the equation for sedentary, overweight individuals presented by Wassermann and Hansen [30]. Heart rate recovery was defined as the difference in heart rate between peak exercise and one-minute later. The ventilatory threshold is the level of oxygen consumption above which aerobic energy production is supplemented by anaerobic mechanisms and was automatically calculated by the V-slope method.

#### Blood pressure, heart rate and biomarkers

Blood pressure was measured in the morning >12 hours after the latest exercise session, in sitting position after 10 minutes’ rest, as the lowest of three consecutive measurements using an oscillometric blood pressure monitor (CARESCAPE V100, GE Healthcare, Horten, Norway). Blood samples were drawn after an overnight fast and analyzed immediately at the hospital laboratory for lipids (low density lipoprotein (LDL), very low-density lipoprotein (VLDL), high density lipoprotein (HDL), P-cholesterol and triglycerides), and endocrine function (HbA1c).

#### Hospital Anxiety and Depression Scale

Anxiety and depression symptoms were evaluated by the self-assessed questionnaire, the Hospital Anxiety and Depression Scale (HADS). Answers separate into anxiety (HADS-A) and depression (HADS-D) sub-scales [31].

#### Myocardial function

Before the TTDE, a standard transthoracic echocardiography was performed including measurements of cardiac function. Selected measurements of cardiac function were determined under hyperaemia. A detailed methods description is found in **S2 File.**

#### Blinding

All baseline assessments were conducted before randomization. Reading of CFVR, and other echocardiographic measures were performed after follow-up with the reader blinded to participant data and to the baseline results. VO_2_max tests at follow-up were blinded to the conducting physiotherapist, who was not committed to the interventional training program.

### Statistics

#### Sample size

The power calculation was based on the primary outcome measures, CFVR and angina burden. Based on mean CFVR of 2.4 in similar patient populations [32, 33], we expected mean CFVR in this population to be 2.4 and wished to detect a possible true difference of 10% between groups, corresponding to a mean change in CFVR of 0.24. The standard deviation of the within individual change was assumed to be 0.3. With power of 0.8 and two-sided significance level of 0.05, twenty-six participants were required in each group and expecting drop-out we aimed at including 60 participants.

SAQ has five subscales each with a score from 1–100. With 26 participants in each group, a two-sided significance level of 0.05, a power of 80 and an assumed standard deviation of 15, we should be able to detect a true difference in mean score between groups of 12 points on each of the five SAQ scales.

#### Statistical method

Categorical data are presented as number (percentage), continuous data as mean (SD) or, if normal distribution could not be assumed, as median (IQR). Student’s paired t-test was used for within-group comparisons of continuous variables. Categorical data were compared with the use of χ^2^-test. For evaluation of associations between variables of interest and the outcome parameter, linear regression analyses were performed for between group comparisons. The outcome parameter was change from baseline adjusted for the baseline value of the outcome parameter. Between-group differences are presented with 95% CI. All analyses were performed in STATA/IC 13.1 (StataCorp LP, College Station TX, USA).

### Approvals

The trial was conducted in accordance with the Helsinki Declaration, approved by The National Committee on Health Research Ethics and The Danish Data Protection Agency, and registered at clinicaltrials.gov (NCT02910154). Prior to inclusion, we collected written informed consent from all participants, based on oral and written information about the study.

## Results

All 30 patients in the intervention group and 26 in the control group attended follow-up (**Fig 1**). Follow-up of last participant was performed October 1^st^, 2018. Loss to follow-up was due to personal reasons (sickness in the family, inconvenience). Baseline median age (IQR) was 65.2 years (57.1; 70.7), median BMI 30.1 kg/m2 (28.4; 32.7) and median CFVR 2.09 (1.93; 2.40). Risk factor burden was high: 63% had a history of hypertension, 68% had dyslipidemia and 15% had diabetes mellitus with no differences between the groups. Participants were well-treated as reflected in baseline blood pressures and cholesterol levels. Distribution of baseline characteristics are shown in **Table 1**.

**Table 1:** Baseline characteristics of participants who completed the study.

	Control (n = 26)	Intervention (n = 30)
**Clinical parameters**		
Age (years)	63.0 (8.0)	64.3 (7.6)
BMI (kg /m2)	29.2 (27.8; 32.3)	30.8 (28.2; 33.7)
SBP (mmHg)	138.3 (18.1)	138.3 (17.2)
DBP (mmHg)	75.7 (7.4)	73.0 (8.1)
HR at rest (bpm)	72.3 (11.5)	67.6 (9.1)
**Anamnestic parameters**		
Family history of cardiovascular disease	16 (64%)	13 (43%)
Cerebral or peripheral artery disease	4 (15%)	4 (13%)
Hypercholesterolemia	19 (73%)	18 (60%)
Hypertension	17 (65%)	17 (57%)
Current smoking	3 (12%)	3 (10%)
Diabetes mellitus type II	6 (23%)	3 (10%)
Duration of diabetes II (years)	8.0 (5.5)	3.5 (2.1)
Diffuse atheromatosis at CAG	7 (35%)	10 (42%)
**Treatment received at baseline**		
Antihypertensives*	15 (58%)	17 (57%)
ACE-inhibitors and ARB	7 (27%)	9 (30%)
Calcium antagonists	8 (31%)	5 (17%)
Betablockers	5 (19%)	9 (30%)
Statins/ezetimibe	14 (54%)	16 (53%)
Metformin	5 (19%)	2 (7%)
Anticoagulants	3 (12%)	3 (10%)
Aspirin	12 (46%)	9 (30%)
Diuretics	9 (35%)	9 (30%)
Nitrates	2 (8%)	4 (13%)

Data are presented as mean (standard deviation), median (interquartile range) or numbers (%). SBP: systolic blood pressure. DBP: diastolic blood pressure. HR: heart rate.

*Antihypertensives: sum of ACE-inhibitors, betablockers, calcium antagonists and ARB: angiotensin II receptor blockers.

Some participants had signs of possible ischaemia on ECG at rest at baseline. Q-waves in the inferior leads were found in six participants. One participant had a left bundle branch block. No ECG abnormalities developed during the intervention period.

### Effect of the intervention on primary outcome measures

#### Coronary microvascular function

Baseline median (IQR) CFVR was 2.09 (1.93; 2.40) with no difference between groups (p = 0.144). There was an insignificant improvement in CFVR in both groups and no between-group difference in CFVR (p = 0.468) **(Table 2).** Weight loss and change in exercise capacity were not correlated to change in CFVR.

**Table 2:** Microvascular function and symptom burden.

	Control Group (N = 26)			Intervention Group (N = 30)			Intervention—Control*	
	Baseline	Follow-up	P	Baseline	Follow-up	P	Difference in change	P
	Mean (SD)	Mean (SD)		Mean (SD)	Mean (SD)		Mean (CI)	
**TTDE Measurements**								
CFVR	2.090 (0.271)	2.237 (0.429)	0.0760	2.198 (0.328)	2.413 (0.654)	0.0693	0.106 (-0.184; 0.395)	0.468
CFV, rest (m/s)	0.296 (0.062)	0.289 (0.056)	0.5575	0.294 (0.064)	0.278 (0.071)	0.2789	-0.010 (-0.043; 0.022)	0.534
CFV, hyperaemia (m/s)	0.609 (0.097)	0.639 (0.142)	0.2414	0.642 (0.167)	0.646 (0.146)	0.8850	-0.014 (-0.077; 0.050)	0.670
**Seattle Angina Questionnaire scale scores**								
Physical limitation	69.7 (22.4)	76.5 (21.5)	0.0696	74.2 (17.8)	82.9 (16.0)	0.0010	3.43 (-4.11; 10.98)	0.364
Angina stability	53.1 (28.3)	54.6 (30.2)	0.7791	52.7 (24.9)	73.3 (26.4)	0.0008	18.91 (5.10; 32.72)	0.008
Angina frequency	74.2 (18.8)	73.5 (19.6)	0.8395	72.0 (20.9)	85.3 (15.3)	0.0002	12.91 (4.91; 20.90)	0.002
Disease perception	60.6 (18.2)	59.9 (25.0)	0.9086	62.5 (20.7)	72.8 (15.2)	0.0058	12.25 (1.73; 22.77)	0.023
Treatment satisfaction	74.5 (23.0)	78.9 (23.1)	0.3885	71.8 (24.2)	87.0 (16.6)	0.0005	9.15 (-0.61; 18.92)	0.066
**Hospital Anxiety and Depression Scale**								
HADS-A	5.8 (3.3)	5.2 (3.2)	0.2164	4.4 (3.2)	3.8 (3.1)	0.1608	0.40 (-1.58; 0.79)	0.507
HADS-D	2.8 (2.7)	2.5 (2.3)	0.5219	2.7 (3.4)	1.3 (1.9)	0.0076	-1.16 (-2.12; -0.22)	0.018

*Between-group differences in change from baseline are adjusted for the baseline value of the dependent variable. Control group is reference. CFV(R): coronary flow velocity (reserve).

#### Angina burden

Mean score was 74 on the angina frequency scale indicating moderate angina:18% reported daily episodes of angina and 26% reported angina less than weekly. At follow-up, Significant between-group differences were seen for the angina stability score, the angina frequency score and the disease perception score **(Table 2).** The intervention group improved markedly on all five SAQ scales (>9 improvement in score). In the control group no changes were seen.

### Effect of the intervention on secondary outcome measures

#### Anxiety and depression

Levels of anxiety and depression symptoms remained low at follow-up. Depression symptoms decreased slightly with a between-group mean difference of 1.16 (2.12; 0.22) points, (p = 0.018). On the anxiety scale, there was no significant in-group or between-group differences. **(Table 2).**

#### Body composition and exercise capacity

Overall, the intervention was effective in achieving weight loss and risk factor control. The intervention resulted in a weight loss of -9.63 (CI: -11.78; 7.80) kg (between group-difference) explained by a decrease in weight in the intervention group). The between-group difference was significant (all p <0.0001) in favor of the intervention group on all body composition parameters except waist hip-ratio and fat free mass (**Table 3**). In the control group, participants obtained a small, insignificant weight loss but a significant decrease in waist circumference (p <0.0001) and in waist hip ratio (p = 0.037).

**Table 3:** Physical fitness, body weight and body composition.

	Control Group (N = 26)			Intervention Group (N = 30)			Intervention—Control*	
	Baseline	Follow-up	P	Baseline	Follow-up	P	Difference in change	P
	Mean (SD)	Mean (SD)		Mean (SD)	Mean (SD)		Mean (CI)	
**Body composition**								
BW (kg)	81.99 (8.9)	82.03 (9.5)	0.9482	84.07 (9.2)	74.43 (9.2)	<0.0001	-9.63 (-11.78; 7.80)	<0.0001
FFM (kg)	45.41 (3.4)	45.50 (3.9)	0.7645	45.50 (4.3)	45.00 (4.8)	0.0461	-0.62 (-1.42; 0.17)	0.120
Waist (cm)	101.7 (9.2)	98.3 (9.6)	<0.0001	101.8 (9.6)	92.2 (10.3)	<0.0001	-6.25 (-8.56; -3.95))	<0.0001
Hip (cm)	112.9 (8.6)	111.8 (8.8)	0.1020	114.7 (7.9)	106.7 (8.5)	<0.0001	-6.78 (-8.67; -4.89)	<0.0001
Waist/hip-ratio	0.898 (0.07)	0.878 (0.07)	0.0371	0.887 (0.07)	0.865 (0.07)	0.0172	-0.004 (-0.03; 0.02)	0.735
**Physical Fitness**								
VO_2_peak (mL/min)	1493.8 (277.9)	1469.2 (215.8)	0.5285	1559.9 (316.0)	1504.7 (364.9)	0.3580	-0.10 (-143.7; 143.5)	0.999
VO_2_peak_BW_ (mL/kg/min)	18.97 (3.5)	18.90 (3.3)	0.8733	18.59 (3.2)	20.05 (4.8)	0.0949	-1.47 (-0.60; 3.53)	0.160
VO_2_peak_FFM_ (mL min/kg FFM^0.67^)	113.4 (16.3)	111.6 (13.1)	0.5795	119.5 (20.3)	116.3 (24.8)	0.5024	2.14 (-9.31; 13.58)	0.708
Max workload (watt)	114 (24)	107.4 (20.3)	0.0629	113 (32)	121.6 (35.0)	0.0095	14.5 (5.03; 23.97)	0.003
Peak RER	1.14 (0.1)	1.15 (0.1)	0.6570	1.11 (0.1)	1.15 (0.08)	0.0233	0.012 (-0.04; 0.06)	0.616

* Between-group differences in change from baseline are adjusted for the baseline value of the dependent variable. Control group is reference. BW: Body weight. FFM: Fat free mass. VO_2_peak: Peak aerobic capacity. RER: Respiratory exchange ratio. SBP: Systolic blood pressure. HR: heart rate.

Baseline mean (SD) VO_2_peak_bw_ was 18.59 (3.2) mL/kg/min with no difference between groups. Twenty-three participants (77%) participated in >50% of training sessions. All exercise tests were terminated due to physical exhaustion. There was no significant change in VO_2_peak or heart rate recovery due to the intervention (between-group p = 0.003) regardless of VO_2_peak being adjusted for change in body weight and fat free mass. Mean workload improved in the intervention group and declined in the control group resulting in a significant between group mean difference at follow-up of 14.5 (CI 5.03; 23.97) watt.

Maximal heart rate remained unchanged in both groups. No exercise test parameters changed in the control group (**Table 3**). Training compliance in the intervention group was not related to exercise capacity at follow-up.

#### Blood pressure, dyslipidemia and glycemic control

Many participants received antihypertensive treatment at the time of inclusion. In the intervention group, five (17%) had antihypertensive treatment initiated as part of the intervention and one (7%) had metformin initiated. Statins were initiated in 11 (37%) from the intervention group. Only one control group participant reported medicine adjustments during the 24 weeks (she initiated statin treatment).

Blood pressure was well-treated in both groups at baseline. At follow-up, there were no between group differences as blood pressure decreased in both groups. In non-diabetes participants, HbA1c decreased in both groups also with no between group difference. In diabetes participants, mean change in HbA1c was different between groups, but results should be interpreted with caution due to few observations. Due to the intervention, total cholesterol decreased 0.55 (0.12; 0.98) mmol/L, (p = 0.014) and triglycerides decreased by 0.52 (0.25; 0.78) mmol/L, (p<0.0001) **(Table 4).** In the intervention group, all lipids except HDL improved. LDL also decreased in the control group.

**Table 4:** Blood pressure and biomarkers.

	Control Group (N = 26)			Intervention Group (N = 30)			Intervention—Control*	
	Baseline	Follow-up	P	Baseline	Follow-up	P	Difference in change	P
	Mean (SD)	Mean (SD)		Mean (SD)	Mean (SD)		Mean (CI)	
**Blood pressure and heart rate**								
SBP (mmHg)	138.3 (18.1)	131.2 (16.3)	0.0087	137.6 (17.1)	131.3 (13.4)	0.0639	0.49 (-6.23; 7.22)	0.884
DBP (mmHg)	75.7 (7.4)	70.5 (10.0)	0.0033	72.8 (8.2)	70.8 (7.7)	0.1510	2.18 (-1.82; 6.19)	0.279
Heart rate (bpm)	70.4 (11.9)	65.7 (11.8)	0.0703	67.2 (10.7)	64.8 (11.0)	0.3673	-0.03 (-6.07; 6.01)	0.991
**Blood biomarkers**								
HbA1c if DM (mmol/mol)	55.0 (20.0)	63.4 (27.0)	0.0886	59.3 (14.4)	40.7 (3.5)	0.2088	-27.1 (-52.6; -1.60)	0.041
HbA1c if no DM (mmol/mol)	37.5 (4.1)	36.4 (2.9)	0.0375	38.0 (2.9)	37.2 (2.5)	0.0294	0.47 (-0.41; 1.33)	0.293
Total cholesterol (mmol/L)	4.9 (0.9)	4.7 (1.0)	0.0721	4.8 (1.1)	4.1 (0.9)	0.0012	-0.55 (-0.98; -0.12)	0.014
LDL cholesterol (mmol/L)	2.6 (0.7)	2.3 (0.8)	0.0474	2.6 (0.9)	2.0 (0.7)	0.0059	-0.30 (-0.69; 0.10)	0.134
HDL cholesterol (mmol/L)	1.6 (0.6)	1.6 (0.5)	0.6926	1.6 (0.4)	1.6 (0.4)	0.4693	0.07 (-0.12; 0.27)	0.457
Triglycerides (mmol/L)	1.5 (0.7)	1.5 (0.8)	0.7345	1.5 (0.9)	1.0 (0.5)	0.0010	-0.52 (-0.78; -0.25)	<0.0001

* Between-group differences in change from baseline are adjusted for the baseline value of the dependent variable. Control group is reference. SBP: Systolic blood pressure. DBP: diastolic blood pressure. HR: heart rate. DM: diabetes mellitus.

#### Myocardial function

Systolic or diastolic myocardial function was normal in the two groups at baseline and only E/A ratio changed significantly at follow-up explained by a slight decrease in the intervention group **(S2 Table).**

## Discussion

The relation between CMD and angina pectoris is not clearly understood. We examined whether an intervention against risk factors for CMD would be feasible and improve symptoms and coronary microvascular function in women with angina and no obstructive CAD. If positive, this would indicate a treatment of microvascular angina and should be followed by larger trials. In this trial, we saw a significant improvement in symptoms with no concomitant improvement in CFVR. The comprehensive intervention was successful in achieving weight loss, optimizing blood pressure and metabolic control and improving lipid profile.

### Coronary microvascular dysfunction

Despite successful weight loss and intensified risk factor control we found no effect on coronary microvascular function. The confidence intervals of mean change in CFVR are relatively narrow, and it is unlikely that we have missed a large effect due to variation or inaccurate measurements of CFVR.

CMD is associated with ageing [5, 34], smoking [35], hypertension [33, 34], atherosclerosis [35], hypercholesterolemia [36], adipositas [37], diabetes [34, 35, 37], and metabolic syndrome [38]. Improvement of non-endothelial dependent microvascular function has been found after risk factor amelioration in studies with similar or even shorter follow-up duration: In a randomized trial of overweight patients with former CAD (primarily men) both weight loss and intensive exercise training significantly improved CFVR [8] however, the trial did not include a control group. Small trials have reported individual effects on CFVR after pharmacological treatment with atorvastatin [39], angiotensin II receptor blockers [40], or angiotensin converting enzyme inhibition [10].

Our findings are supported by other intervention studies on the iPOWER cohort: a controlled study comprising 12 weeks of liraglutide treatment resulted in a mean weight loss of 6 kg (7%) but in no change in CFVR [41]. In a randomized, placebo-controlled study comprising 24 weeks of treatment with angiotensin converting enzyme inhibitor to normotensive participants, there was no effect on CFVR [42]. In a recent intervention study of patients with newly diagnosed myocardial infarction, six months’ treatment with rosuvastatin had no effect on invasively assessed coronary microvascular function [43]. Thus, trials showing effect of intervention on non-endothelial dependent coronary vascular function are lacking. Importantly, in this study, we assessed the non-endothelial dependent coronary microvascular function. It is possible that the intervention would have shown improvement in endothelial dependent vascular function, and indeed there is much evidence in support of an effect of risk factor control on peripheral endothelial function [9, 11, 44, 45].

### Angina burden

According to expert consensus, the diagnosis of MVA requires symptoms of myocardial ischaemia, absence of obstructive CAD, evidence of CMD, and evidence of ischaemia by non-invasive stress tests [46]. CMD is a flow limiting condition. Thus, it is reasonable, that MVA is primarily characterized as exercise induced similar to stable angina due to obstructive CAD [12, 47]. However, differences in pain characteristics between MVA and macrovascular CAD occur, and longer pain duration, symptoms at rest, dyspnea and poor effect of nitrates may be more common in MVA [12]. Although MVA may present with varying symptomatology, baseline angina burden in this study was comparable to that of angina patients with obstructive CAD in the ISCHEMIA trial [48], the ORBITA [49] and the COURAGE [50] trials. The comprehensive intervention in this trial resulted in clinically relevant improvement in angina frequency, stability and disease perception on SAQ scales. In the ISCHEMIA trial, the invasive strategy group had a mean improvement in SAQ summary score of 4.2 points at the one-year follow-up [48]. The ORBITA trial [49] compared percutaneous coronary intervention with a placebo intervention and found a change from baseline in SAQ score of 4–11 with no difference between treatment groups. Thus, the improvement in angina burden observed in this study is comparable to that achieved with revascularization, regardless of whether it was due to placebo, a consequence of the obtained weight loss or an effect of being observed, as discussed in the limitations section [51].

Indisputably, improvement of MVA is important from a clinical perspective, and may increase quality of life and decrease morbidity due to repeated cardiac catheterization in patients with no obstructive CAD [13, 52]. Evidence of improvement of MVA requires large, randomized, clinical trials examining the effect of intervention on definite MVA, however such studies are still lacking. In suspected MVA, common anti-anginal therapy have shown to be effective treatment and count betablockers, nitrates, calcium channel blockers, and ranolazine [13, 53–57]. Statins and aspirin may decrease endothelial inflammation and atherosclerosis, leading to improved endothelial dependent coronary microvascular function and indirectly improved MVA [58]. Patients with MVA may have different treatment preferences and manifestations of symptoms. Thus, treatment must be individually tailored and based on the patient’s risk factors and suspected pathophysiology of CMD [54].

### Anxiety and depression

Although mean scores of anxiety and depression were low at baseline, symptoms of depression improved significantly, but modestly. This result is supported by a systematic review and meta-analysis that found overall improvement in depression symptoms after weight loss interventions [59]. The CUT-IT trial showed similar improvement in HADS depression score at 12-week follow-up [22].

### Weight loss and body composition

We aimed at a 10% weight loss in the intervention group and obtained a weight loss of 11.5%, primarily from body fat. We succeeded in achieving this weight loss without significant loss of muscle mass, possibly because of the simultaneous exercise program. There were minor side-effects to the LED diet (borborygmi, headache and small sodium and potassium fluctuations during the first 2–4 weeks) but no serious adverse effects.

### Exercise capacity

Mean exercise capacity was lower than expected from sedentary, overweight women [30]. This has also been shown in a previous sub-study of women recruited from the iPOWER cohort [60]. Maximal workload improved in the intervention group and declined in the control group, but this improvement did not translate into an improvement in VO_2_peak, nor did it result in improved HR recovery or in ventilatory threshold as indicators of improved fitness and work economy, respectively. We find it difficult to reconcile these findings but speculate that the adjustment for body-mass does not fully normalize exercise capacity to body composition in a situation with large weight changes. The lack of increase in exercise capacity can also be due to the severely reduced calorie intake during the first 12 weeks of intervention, which might have hampered performance and ability to improve physical fitness. However, there was only a slight loss of muscle mass despite the large weight loss.

Inadequate training compliance was also a concern. Several participants lived at a distance from the hospital and found it difficult to participate in at least 50% of the training sessions. With only two supervised training sessions weekly, the study was vulnerable to non-compliance to improve exercise capacity. Nevertheless, registered training compliance was not related to change in exercise capacity.

### Blood pressure, dyslipidemia and glycemic control

Blood pressure declined over the course of the study with no between group differences. However, most participants were well-treated before inclusion which could explain the lacking treatment response. Total cholesterol, LDL and triglycerides improved in the intervention group, presumably due to weight loss and more intensive statin treatment. The small decrease in LDL cholesterol, HbA1c and blood pressure observed in the control group may be explained by a modest weight loss and diet changes in these participants also.

### Strengths and limitations

Major strengths of this study are the randomized design, no dropouts from the intervention group and the systematic inclusion of patients from well-defined criteria. However, the study has several limitations. Participants were generally well-treated for conditions that are normally associated with CMD. Individual differences in improvement of exercise capacity, weight loss and CFVR challenged our results, and the sample size was too small for subgroup analyses and increases the risk of type II error (e.g. missing a true effect of the intervention on CFVR). Moreover, the drop-out rate in the control group was larger than expected. We assume that the compliance to the study was driven by motivation, and that the willingness to come to follow-up, despite the inconvenience it may have caused, was dominant in the intervention group.

As shown in the ORBITA trial, symptoms are very susceptible to placebo. We cannot rule out that the observed improvement in angina burden is a placebo effect. Similar criticism has been directed at the ISCHEMIA trial and other trials with no blinding of the intervention. Moreover, symptom reporting may have been influenced by a Hawthorne effect, which is an effect of being observed. The Hawthorne effect might influence participants in both randomization groups [51]. We tried to minimized this effect by not observing participants while they filled out the questionnaires, and by collecting the questionnaires without looking at the answers until the end of study. However, blinding of participants was impossible because of the study design, thus a Hawthorne effect is a challenge in lifestyle intervention studies.

Finally, improvement in symptoms may be related to improvement in endothelial dependent vascular function, which was not assessed in this trial.

## Conclusion

A comprehensive risk factor intervention including a major weight loss resulted in significant improvement in angina burden but no improvement of coronary microvascular function among overweight women with microvascular angina and had no side effects.

Individually tailored intervention may be effective in the treatment of CMD and MVA due to heterogeneity in symptoms, treatment preferences, personal resources, pathophysiology and risk factors for CMD.

## Supporting information

S1 Fig Participants’ study plan. Study time plan and elements of the intervention. The plan is individualized and applicable to any participant regardless of randomization group or time of inclusion.
(TIF)

S2 Fig Antihypertensive treatment algorithm. Intervention group participants with hypertension, hypercholesterolemia or diabetes are enrolled in the medication plan according to criteria defined in S1 Table.
(TIF)

S1 Table Components of the 24-week intervention. Thresholds for medical treatment and treatment goals of the 24-week intervention.
(DOCX)

S2 Table Transthoracic echocardiography. Results of parameters of interest from the standardized echocardiographic examination performed at baseline and follow-up.
(DOCX)

S1 File CONSORT 2010 checklist of information to include when reporting a randomised trial*. (DOC)

S2 File Methods description of TTE. Description of the standardized transthoracic echocardiography performed in all participants before the CFVR examination. Some measurements were repeated at hyperemia.
(DOCX)

S3 File (DOCX)

## Abbreviations

AIT: Aerobic interval training
BMI: Body mass index
CAD: Coronary artery disease
CAG: Coronary arteriography
CFV(R): Coronary flow velocity (reserve)
CMD: Coronary microvessel dysfunction
DEXA: Dual X-ray absorptiometry array
HADS: Hospital Anxiety and Depression Scale
LED: Low energy diet
LVEF: Left ventricular ejection fraction
RER: Respiratory exchange ratio
SAQ: Seattle angina questionnaire
TTDE: Transthoracic Doppler stress echocardiography
