# Supplementary figures and images for: Comprehensive treatment of microvascular angina in overweight women – a randomized controlled pilot trial
Source: PLoS One. 2020 Nov 5;15(11):e0240722. doi: 10.1371/journal.pone.0240722 (PMC7644075; doi:10.1371/journal.pone.0240722)

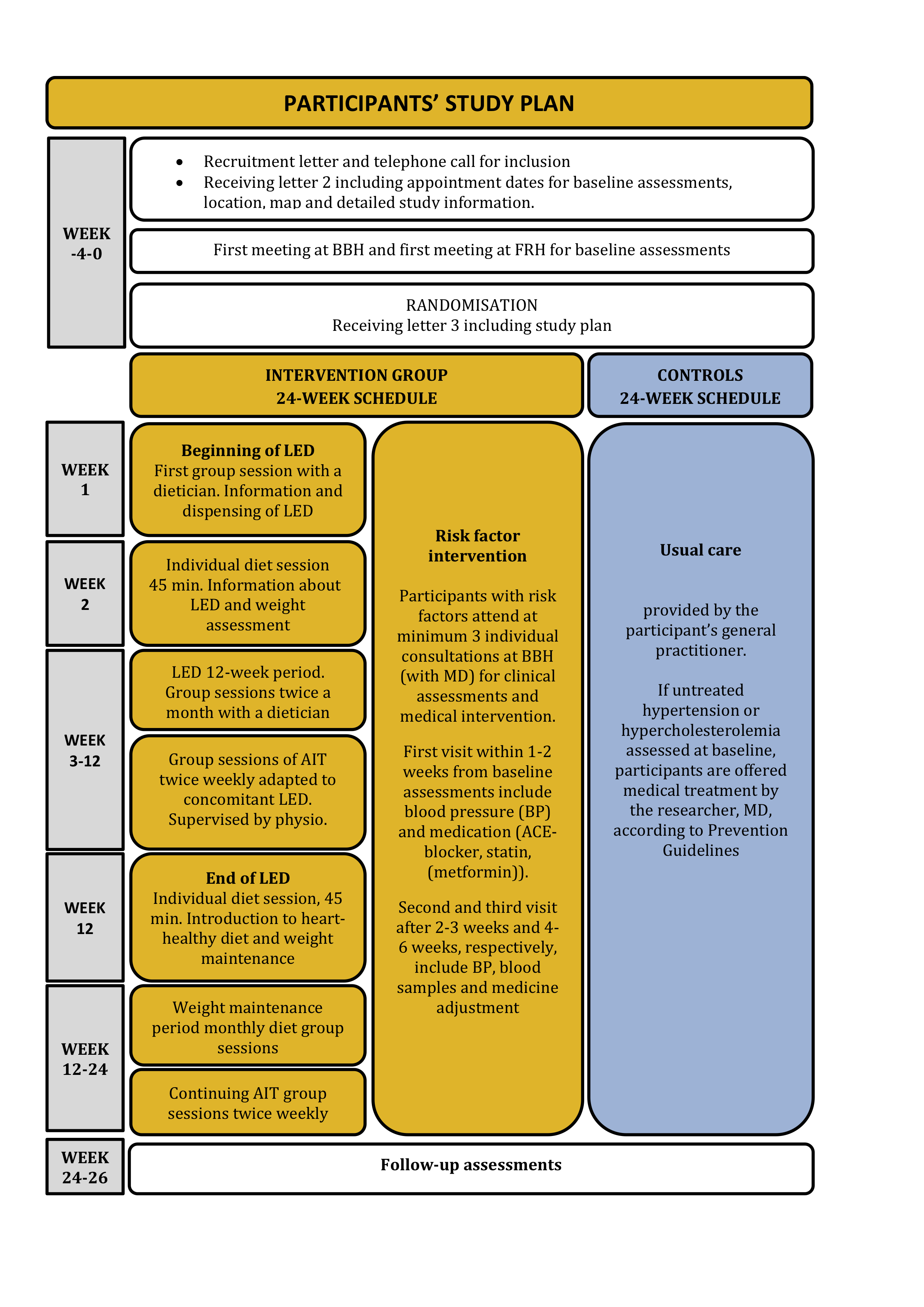

Supplement: S1 Fig — Study time plan and elements of the intervention. The plan is individualized and applicable to any participant regardless of randomization group or time of inclusion. (TIF) [file pone.0240722.s001.tif]

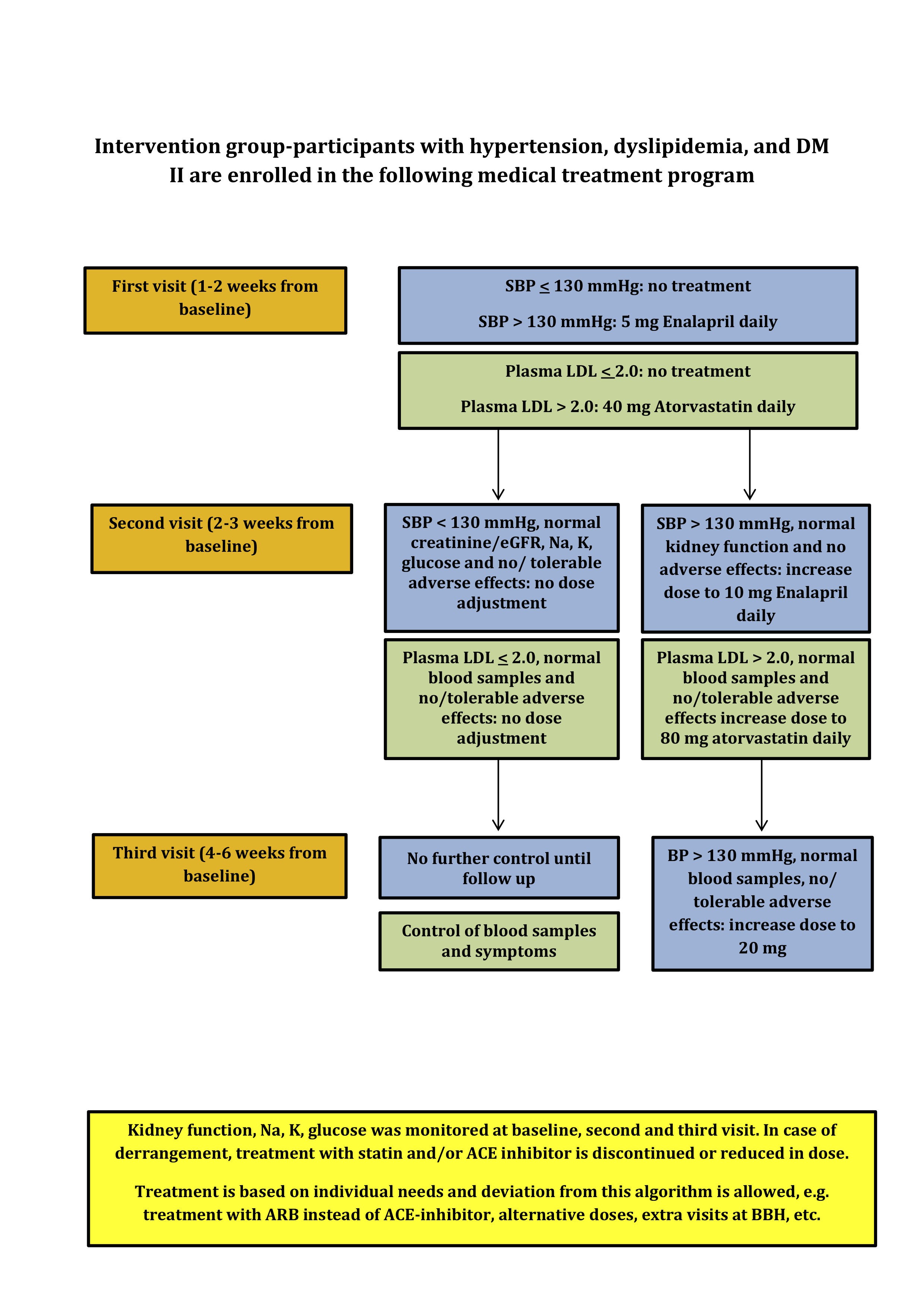

Supplement: S2 Fig — Intervention group participants with hypertension, hypercholesterolemia or diabetes are enrolled in the medication plan according to criteria defined in S1 Table. (TIF) [file pone.0240722.s002.tif]
