# Supplementary material for: Comprehensive treatment of microvascular angina in overweight women – a randomized controlled pilot trial
Source: PLoS One. 2020 Nov 5;15(11):e0240722. doi: 10.1371/journal.pone.0240722 (PMC7644075; doi:10.1371/journal.pone.0240722)
Supplement: S1 Table — Thresholds for medical treatment and treatment goals of the 24-week intervention. (DOCX) [file pone.0240722.s003.docx]

## S1 Table. Components of the 24-week intervention

|  | Target group | Duration | Intervention | Sessions | Goal | Responsible |
| --- | --- | --- | --- | --- | --- | --- |
| Aerobic exercise training (AIT)  Week 0-24 | All | 1½ h | Twice a week | Group | VO_2_ peak  10% improved | Card. Rehab Team: Physiotherapist |
| Weight loss  Week 0-12 | All | 1 h | Every second week +  1 individual | Group +  individual | 10% weight loss | Card. Rehab Team: Dietician |
| Weight maintenance  Week 12-24 | All | 1 h | Monthly +  1 individual | Group +  individual | Heart healthy diet | Card. Rehab Team: Dietician |
| Optimized medication | P-LDL > 2.0  SBP >130  HbA1c >6.0 | ½ h | Statin  ACE inhibitor  Metformin | Individual | LDL < 2.0  BP 130/80  HbA1c < 6.5 | Medical Doctor |

VO_2_peak: Peak aerobic capacity. LDL: low density lipoprotein. (S)BP: (systolic) blood pressure. HbA1c: glycosylated hemoglobin. ACE : angiotensin converting enzyme.
