# Supplementary material for: Comprehensive treatment of microvascular angina in overweight women – a randomized controlled pilot trial
Source: PLoS One. 2020 Nov 5;15(11):e0240722. doi: 10.1371/journal.pone.0240722 (PMC7644075; doi:10.1371/journal.pone.0240722)
Supplement: S2 Table — Results of parameters of interest from the standardized echocardiographic examination performed at baseline and follow-up. (DOCX) [file pone.0240722.s004.docx]

## S2 Table. Transthoracic echocardiography

|  | Control Group (N=26) | | |  | Intervention Group (N=30) | | |  | Intervention - Control* | |
| --- | --- | --- | --- | --- | --- | --- | --- | --- | --- | --- |
|  | Baseline | Change | P |  | Baseline | Change | P |  | Difference in Change | P |
|  | Mean (SD) | Mean (CI) |  |  | Mean (SD) | Mean (CI) |  |  | Mean (CI) |  |
| Systolic heart function | | | | | | | | | | |
| LVEF, rest (%) | 58.50 (5.27) | 0.43 (-1.97; 2.83) | 0.7149 |  | 60.46 (4.82) | -1.61 (-3.50; 0.28) | 0.0914 |  | 1.64 (-0.90; 4.19) | 0.200 |
| LVEF, hyperaemia (%) | 60.52 (4.65) | -0.99 (-3.17; 1.19) | 0.3556 |  | 62.11 (4.53) | -1.16 (-3.04; 0.71) | 0.2140 |  | 0.95 (-1.29; 3.19) | 0.398 |
| GLS peak-systolic, rest (%) | 20.79 (3.22) | -0.16 (-1.34; 1.02) | 0.7786 |  | 21.57 (2.73) | -0.31 (-1.41; 0.79) | 0.5656 |  | 0.45 (-0.96; 1.86) | 0.521 |
| GLS peak-systolic, hyperaemia (%) | 22.94 (3.29) | -0.44 (-2.17; 1.29) | 0.6003 |  | 23.14 (3.04) | -0.92 (-2.03; 0.19) | 0.1011 |  | 0.31 (-1.21; 1.84) | 0.681 |
| LVMI (mg/m2) | 86.09 (17.06) | -4.29 (-11.33; 2.74) | 0.2206 |  | 81.57 (19.87) | 5.63 (-11.57; 0.32) | 0.0627 |  | 0.84 (-9.36; 7.69) | 0.845 |
| Diastolic heart function |  |  |  |  |  |  |  |  |  |  |
| LAVI (mL/m2) | 28.60 (6.20) | -1.05 (-3.27; 1.16) | 0.3364 |  | 30.41 (8.07) | -2.14 (-4.08; 0.20) | 0.0315 |  | 1.20 (-1.64; 4.04) | 0.401 |
| Deceleration time (ms) | 197.54 (32.86) | 4.81 (-9.94; 19.55) | 0.5082 |  | 208.10 (33.48) | -3.24 (-17.28; 10.81) | 0.6394 |  | 6.77 (-9.89; 23.43) | 0.418 |
| E/A ratio | 1.00 (0.22) | 0.02 (-0.06; 0.09) | 0.6493 |  | 1.07 (0.25) | -0.10 (-0.17; 0.04) | 0.0028 |  | 0.11 (0.02; 0.20) | 0.022 |
| e' (cm/s) | 8.84 (2.00) | -0.36 (-1.04; 0.31) | 0.2775 |  | 9.13 (2.14) | -0.33 (-0.96; 0.31) | 0.2972 |  | 0.07 (-0.68; 0.83) | 0.844 |
| E/e' ratio | 9.86 (2.70) | 0.47 (-0.55; 1.50) | 0.3503 |  | 9.92 (2.45) | 0.29 (-0.43; 1.01) | 0.4098 |  | 0.14 (-0.91; 1.19) | 0.795 |

* Between-group differences in change from baseline are calculated by linear regression adjusted for the baseline value of the dependent variable. Control group is reference. LVMI: left ventricular mass index. LAVI: Left atrial volume indexed to Body Surface Area (BSA). E: Early mitral inflow velocity. e’: mitral annular early diastolic velocit
