# Supplementary material for: Comprehensive treatment of microvascular angina in overweight women – a randomized controlled pilot trial
Source: PLoS One. 2020 Nov 5;15(11):e0240722. doi: 10.1371/journal.pone.0240722 (PMC7644075; doi:10.1371/journal.pone.0240722)
Supplement: S2 File — Description of the standardized transthoracic echocardiography performed in all participants before the CFVR examination. Some measurements were repeated at hyperemia. (DOCX) [file pone.0240722.s006.docx]

# S2 File. Methods description of transthoracic echocardiography

A standard transthoracic echocardiography was performed using GE Healthcare Vivid E9 cardiovascular ultrasound system (GE Healthcare, Horten, Norway) and a 1.3-4.0 MHz transducer (GE Vivid 5S probe). Global longitudinal strain (GLS) was determined using software for speckle tracking analyses (Q-analysis, GE EchoPAC v112, Norway).

We acquired 2-dimensional images of the left ventricle (LV) in apical long axis, 2- and 4-chamber views at frame rates between 60-90 frames/s adjusted to the patient’s heart rate. Left ventricular ejection fraction (LVEF) was analyzed as a semi-automated biplane calculation (Auto-EF tool, GE EchoPAC v112). Measurements of left ventricular internal dimensions, left ventricle mass index and left atrium volume index by the Volume Methods of Discs were performed and calculated according to European and American recommendations (1–3). Aortic valve closure was defined in tissue Doppler M-mode. GLS was calculated as the average of all accepted segmental values of peak systolic strain (4). Only 3 discarded segments were permitted.

Assessment of diastolic function was based on recommendations from the American and European Society of Echocardiography for 2009 (5). The updated recommendations from 2016 were not applicable as we have not assessed the tricuspid regurgitation velocity in this study (1). Mitral inflow velocities, the E/A ratio, and tissue Doppler of an average of late diastolic velocities in the mitral septal and lateral annulus, e’, were used as surrogate markers of diastolic relaxation and left ventricular compliance. Deceleration time was used as a surrogate of early left ventricular stiffness; E/e’ as a surrogate estimate of left ventricular filling pressures and left atrial volume index as a measure of increased left ventricular filling pressure. We also acquired two-dimensional images of the LV in apical long axis, 2- and 4-chamber views during adenosine infusion to assess LVEF and global longitudinal strain under hyperaemia. Images were stored for offline analysis.

# References

1. Nagueh SF, Smiseth OA, Appleton CP, Byrd BF, Dokainish H, Edvardsen T, et al. Recommendations for the Evaluation of Left Ventricular Diastolic Function by Echocardiography: An Update from the American Society of Echocardiography and the European Association of Cardiovascular Imaging. J Am Soc Echocardiogr. Elsevier Inc; 2016;29(4):277–314.

2. Lang RM, Badano LP, Mor-Avi V, Afilalo J, Armstrong A, Ernande L, et al. Recommendations for cardiac chamber quantification by echocardiography in adults: an update from the American Society of Echocardiography and the European Association of Cardiovascular Imaging. J Am Soc Echocardiogr. Elsevier; 2015 Jan 1;28(1):1–39.e14.

3. Barbieri A, Bursi F, Mantovani F, Valenti C, Quaglia M, Berti E, et al. Left ventricular hypertrophy reclassification and death: application of the Recommendation of the American Society of Echocardiography/European Association of Echocardiography. Eur Hear J - Cardiovasc Imaging. Oxford University Press; 2012 Jan 1;13(1):109–17.

4. Voigt J-U, Pedrizzetti G, Lysyansky P, Marwick TH, Houle H, Baumann R, et al. Definitions for a common standard for 2D speckle tracking echocardiography: consensus document of the EACVI/ASE/Industry Task Force to standardize deformation imaging. Eur Hear J - Cardiovasc Imaging. Oxford University Press; 2015 Jan 1;16(1):1–11.

5. Nagueh SF, Smiseth OA, Appleton CP, Byrd Iii BF, Dokainish H, Edvardsen T, et al. Recommendations for the Evaluation of Left Ventricular Diastolic Function by Echocardiography: An Update from the American Society of Echocardiography and the European Association of Cardiovascular Imaging.
