# Supplementary material for: Comprehensive treatment of microvascular angina in overweight women – a randomized controlled pilot trial
Source: PLoS One. 2020 Nov 5;15(11):e0240722. doi: 10.1371/journal.pone.0240722 (PMC7644075; doi:10.1371/journal.pone.0240722)
Supplement: S3 File — (DOCX) [file pone.0240722.s007.docx]

**Protocol – CORA**

**Title** Comprehensive treatment of angina in women with microvascular dysfunction– a ‘proof of concept’ study of the iPOWER cohort (CORA)

**Date** 27.11.2016

**Investigator** Kira Bang Bové, MD

Department of Cardiology, Bispebjerg University Hospital

Bispebjerg Bakke 23, 2400 Copenhagen NV, Denmark

**Supervisor** Eva Prescott, MD, DMSc

Chief of Cardiovascular Research

Department of Cardiology, Bispebjerg University Hospital

Bispebjerg Bakke 23, 2400 Copenhagen NV, Denmark

**Primary co-supervisor** Arne Astrup, MD, DMSc

**Co-supervisor** Lene Rørholm Pedersen, MD, PhD

*The study will be conducted in accordance with this protocol, the guidelines for good clinical practice and current regulations by the authorities.*

*Signatures:*

30.03.2016
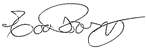
 30.03.2016

___________________________________ __________________________________________

*Date Eva Prescott, professor, MDSc Date Kira Bang Bové, MD*

**Table of contents**

[Abbreviations 4](#_Toc52996236)

[Background 6](#_Toc52996237)

[Rationale for intervention 7](#_Toc52996238)

[Hypothesis 8](#_Toc52996239)

[Aims 8](#_Toc52996240)

[Study design 8](#_Toc52996241)

[Randomization 8](#_Toc52996242)

[Figure 2. Flowchart of the study 9](#_Toc52996243)

[Table 1. Schematic overview of data assessments 10](#_Toc52996244)

[Primary endpoint 10](#_Toc52996245)

[Secondary endpoints 10](#_Toc52996246)

[Figure 3. Project plan milestones 11](#_Toc52996247)

[Study participants 11](#_Toc52996248)

[Inclusion criteria 11](#_Toc52996249)

[Exclusion criteria 11](#_Toc52996250)

[Withdrawal criteria 12](#_Toc52996251)

[Methods 12](#_Toc52996252)

[Blinding 13](#_Toc52996253)

[Intervention 13](#_Toc52996254)

[Table 2. Overview of the components of the 24-week intervention 14](#_Toc52996255)

[Figure 4. Participants’ study plan 15](#_Toc52996256)

[Figure 5. Antihypertensive treatment algorithm 16](#_Toc52996257)

[Exercise training (ET) 17](#_Toc52996258)

[Figure 6. Overview of exercise training group sessions 17](#_Toc52996259)

[Low energy diet (LED) 17](#_Toc52996260)

[Figure 7. Overview of diet sessions 19](#_Toc52996261)

[Controls – usual care 19](#_Toc52996262)

[Description of procedures 19](#_Toc52996263)

[Coronary flow velocity reserve (CFVR), systolic and diastolic function: 19](#_Toc52996264)

[Echocardiography – strain 20](#_Toc52996265)

[Exercise capacity (VO_2_ peak) 20](#_Toc52996266)

[Dexa scan and body composition 20](#_Toc52996267)

[Clinical examination 21](#_Toc52996268)

[Questionnaires: 21](#_Toc52996269)

[Seattle Angina Questionnaire - SAQ 21](#_Toc52996270)

[Hospital Anxiety and Depression Scale – HADS 22](#_Toc52996271)

[Biomarkers 22](#_Toc52996272)

[Biological material 22](#_Toc52996273)

[Statistics 23](#_Toc52996274)

[Sample size 23](#_Toc52996275)

[Risks and disadvantage for participants 24](#_Toc52996276)

[Ethical aspects 25](#_Toc52996277)

[Regulatory authorities 25](#_Toc52996278)

[Informed consent 25](#_Toc52996279)

[Data storage 25](#_Toc52996280)

[Organization of the study 26](#_Toc52996281)

[Costs 27](#_Toc52996282)

[Table 3. Overview of estimated costs of the 24-week intervention 27](#_Toc52996283)

[Table 4. Total costs of the research project 28](#_Toc52996284)

[Publication of results 28](#_Toc52996285)

[References 29](#_Toc52996286)

# Abbreviations

ET Exercise training

ACE Angiotensin converting enzyme

ARB Angiotensin receptor blocker

BMI Body mass index

BP Blood pressure

CAD Coronary artery disease

CAG Coronary arteriography

CI Confidence interval

CPET Coronary-pulmonary exercise test

CFVR Coronary flow velocity reserve

CMD Coronary microvessel dysfunction

DEXA Dual X-ray absorptiometry array

FEV1 Forced expiratory volume first second

FMD Flow mediated (vaso)dilation

HADS Hospital Anxiety and Depression Scale

LAD Left anterior artery

LED Low energy diet

LVEF Left ventricular ejection fraction

NBV Nationale behandlingsvejledning (Dansk Cardiologisk Selskab)

NO Nitric oxide

NTG Nitroglycerine

RER Respiratory exchange ratio

SAQ Seattle angina questionnaire

SD Standard deviation

STEMI ST-elevation myocardial infarction

NSTEMI non-ST-elevation myocardial infarction

TTDSE Transthoracic Doppler stress echocardiography

# Background

Angina pectoris is the most common symptom of coronary heart disease among women but unlike men most women do not have stenosis of the coronary arteries.^1^ ^2^ ^3^ ^4^ Recent studies emphasize that contrary to previous understanding many of these women have a poor

prognosis.^5^ We have recently confirmed this in a Danish population where two thirds of

women referred to invasive angiography had no obstructive coronary disease. This

proportion has increased in the last decade and is associated with frequent hospital readmission, continued angina, loss of quality of life and a five times higher risk of

premature exit from the work force than the background population.^4^ ^6^ ^7^ Thus, there are considerable financial and human costs associated with non-obstructive angina in

women.^8^ Mean age at debut is 57 years^4^ and with an estimated 40.000 women in Denmark

suffering from the condition the benefits from improved care are substantial. Through

clinical and pathological studies microvessel disease as a cause of ischemia, angina and

poorer prognosis has emerged.^9^ ^10^ ^11^

*Microvessel disease*

In the heart 95% of the blood flow is controlled by the microcirculation, **Figure 1.**When oxygen demand is increased the normal response of the microvessels is to reduce resistance in order to increase flow. When microvessels are dysfunctional the blood flow in the larger coronary vessels does not increase sufficiently to meet oxygen demand, thus leading to ischemia and pain. The main causes are thought to be dysfunction of endothelium and structural changes such as perivascular fibrosis and changes in vascular smooth muscle cells. In addition to vasodilation the endothelium plays a central role in the atherosclerotic process by generating vasoactive and anticoagulant factors that are important mediators of thrombosis. Coronary microvessel dysfunction (CMD) has been shown to be a strong predictor of poor cardiovascular prognosis in a wide group of cardiac patients.^12^ ^13^ ^14^ ^15^ ^16^ ^17^

*Proof of concept*

Although microvessel disease is thought to be the cause of angina in a large proportion of patients, CMD is also demonstrable in asymptomatic individuals with cardiovascular risk factors. Data derived from eastern Denmark indicate that more than 50% of women with angina have CMD^4^ but comparative studies of the prevalence of CMD in asymptomatic individuals have not been performed. Thus, CMD may merely be an innocent bystander related to the presence of cardiovascular risk factors rather than the cause of angina symptoms. Furthermore, only a handful of interventional studies have simultaneously addressed CMD and symptoms in this patient group and with diverging results.^18^ ^19^ Thus there is a need of studies showing ‘proof-of-concept’, i.e. that improvement of CMD also improves angina symptoms in these patients.

**Figure 1:**

Photo illustrating extent of coronary microvasculature in relation

to epicardial vessels. Brought with permission from Prof. Camici.

### Rationale for intervention

In women with angina and no obstructive stenosis of the coronary vessels cardiovascular risk factors are common. Among 3000 Danish women with angina and open arteries, 12% had diabetes, 48% hypertension, 20% were smokers and the mean body mass index (BMI) was 27 kg/m^2^.^4^ In a randomized trial among overweight patients with coronary artery disease (CAD) we have recently found that both a large weight loss and intensive exercise training significantly improve coronary flow velocity reserve (CFVR).^20^ ^21^ Small studies addressing risk factors individually suggest an effect on peripheral vascular function of exercise training,^22^ ^23^ statin therapy, and weight loss.^24^ ^25^ Eighty percent of women with CMD have pre-diabetes, which is strongly associated with microvessel disease. Lifestyle intervention significantly reduces risk of developing diabetes.^26^ Medical treatment targeting microvessel dysfunction in patients with angina has not been systematically tested but small studies indicate an effect of beta-blockers ^27^ and Angiotensin Converting Enzyme (ACE)-inhibition^18^ on coronary microvessel function.^24^ Thus, small studies indicate effect of individual interventions but mainly on the function of peripheral vessels. A comprehensive intervention simultaneously targeting CMD and angina has not previously been attempted. The rationale for this present intervention is to test this concept in women with angina and CMD.

### Hypothesis

CMD is the cause of angina pectoris in a large proportion of women with angina. Intervention that improves CMD also leads to amelioration of symptoms and improved prognosis.

# Aims

The aim of this study is a ‘proof-of-concept’, i.e. to determine whether a comprehensive

intervention is feasible and results in improvement in both angina and CMD.

The study is a pilot study which we, if successful, will seek to expand to a multicenter interventional trial with prognostic outcome in collaboration with an international

consortium.

# Study design

The study is a randomized, controlled intervention trial. Patients from the iPower cohort fulfilling the inclusion criteria of this present study are invited to participate. Please see **figure 2** for a schematic overview of the inclusion. Details regarding the study information and informed consent are further described in the chapter “Ethical aspects”. Sixty participants are included in the study. After baseline examinations participants are randomized (1:1) to either comprehensive intervention for 24 weeks or usual care (controls). Baseline and 24-week assessments are performed at the Hospitals Bispebjerg and Frederiksberg. Please see **Table 1.**

## Randomization

A third party unrelated to the study will perform randomization of participants with randomly permuted block sizes of 2,4, and 6 using the ralloc program in Stata 11. Replacement of participants will be possible in case of dropout.

## Figure 2. Flowchart of the study

## Table 1. Schematic overview of data assessments

| **Table 1** | Time (min) | **Baseline**  **Week 0** | | | **Follow-up**  **Week 24** | | |
| --- | --- | --- | --- | --- | --- | --- | --- |
|  |  | stud med | MD | physio | stud med | MD | physio |
| Informed consent | 10 | x |  |  |  |  |  |
| In- and exclusion criteria |  |  |  |  |  |  |  |
| General health examination |  |  |  |  |  |  |  |
| Medical history | 10 | x |  |  | x |  |  |
| ECG | 10 | x |  |  | x |  |  |
| Blood pressure, heart rate, BMI, anthropometry, ECG | 15 | x |  |  | x |  |  |
| Blood samples |  |  |  |  |  |  |  |
| Haematology, lipids, liver, kidney and endocrine function (TSH, glucose, hbA1c, inflammatory markers) | 10 | x |  |  | x |  |  |
| Questionnaires |  |  |  |  |  |  |  |
| HADS | 10 | x |  |  | x |  |  |
| SAQ | 10 | x |  |  | x |  |  |
| Ultrasound tests |  |  |  |  |  |  |  |
| Echocardiography (TTDE) | 40 |  | x |  |  | x |  |
| Coronary flow reserve (CFVR) | 60 |  | x |  |  | x |  |
| Stress echocardiography | 20 |  | x |  |  | x |  |
| Fat distribution and physical test |  |  |  |  |  |  |  |
| DEXA scan | 40 | x |  |  | x |  |  |
| VO_2_max test | 60 |  |  | x |  |  | x |
| Time consumption (hours) |  | ~5 | | 1 |  | | 1 |

## Primary endpoint

• Changes in coronary microvessel function assessed by a transthoracic Doppler echo (TTDE) measured CFVR

## Secondary endpoints

• Changes in symptom burden assessed by the Seattle Angina Questionnaire (SAQ)

• Changes in biomarkers including inflammatory markers and markers of metabolism

• Changes in exercise capacity (VO_2_peak)

• Changes in body weight and fat distribution (DEXA scan)

• Changes in level of anxiety and depression (HADS questionnaire)

• Changes in systolic and diastolic heart function at rest and during stress including advanced imaging (eg. Strain-Echocardiography)

## Figure 3. Project plan milestones

## Study participants

### Inclusion criteria

Patients will be recruited and included from the ongoing iPower study (please see figure 2)^28^ i.e.

- - Female gender
  - 40-75 years of age
  - Referred to a cardiac centre for assessment with coronary angiography due to chest pain or other signs of ischemia leaving out ST-segment elevation myocardial Infarction (STEMI) or NSTEMI patients (elevated enzymes, electrocardiographic (ECG) changes/no ECG changes)
  - No significant stenotic lesions at the following CAG defined as > 50% stenosis of epicardial vessels. Patients are included from the PATS database within 1 year after coronary angiography.
- Angina with a symptom burden of symptoms > monthly
- Impaired coronary microvascular function, defined as a TTDE measured CFVR < 2.5 with a good quality (quality index > 3)
- BMI > 25 (for BMI < 26 waist hip ratio must be > 0.8)
- Informed consent

### Exclusion criteria

- Previously verified MI, verified in medical records: STEMI, elevated coronary markers^2^ or NSTEMI
- Previous percutaneous coronary intervention or coronary artery bypass graft.
- Left ventricular ejection fraction (LVEF) < 45% assessed by echocardiography within 6 months before inclusion
- Any allergies to the content of the LED (gluten/nuts), allergy to dipyridamole, adenosine, or teofyllamine
- Significant valvular heart disease^[[1]](#footnote-1)^
- Congenital heart disease
- Severe asthma
- Severe chronic obstructive pulmonary disease (COPD): FEV1 < 50% of predicted (age, height, ethnicity)
- Severe comorbidity with limited life-expectancy < 1 year
- Chest pain with a strongly suspected non-ischemic etiology (e.g. pericarditis, pneumonia)
- Pregnancy
- Active cancer
- Severe renal failure (eGFR < 30) or severe hepatic comorbidity
- Chronic alcohol abuse
- Atrial flutter or fibrillation
- Atrioventricular block > 1^st^ degree
- Diabetes Mellitus type I
- Participation in other trials if relevant for the present study
- Language- or other barrier to giving informed consent
- Physical or mental disabilities contraindicating or hampering diet or exercise training
- Travel distance to research hospital requiring more than 3 hours of travel, making it difficult for the patient to participate

### Withdrawal criteria

- Sudden unexpected serious adverse reaction or sustained side effects
- Poor compliance with LED, training or medicine compliance defined in the methodology chapter.

# Methods

Sixty participants are included, examined at baseline and randomized (1:1) to either comprehensive treatment or usual care (controls). Comprehensive treatment consists of 24 weeks of LED, exercise training and optimized medical treatment. Usual care is medical treatment handled by the researcher, MD, at baseline and follow-up. In case of delay, e.g. cancellation of appointments by the participants, four weeks are accepted between baseline assessments and start of intervention (week 0, defined by the first (baseline-) meeting with dietician). A delay of up to four weeks (eg. cancellation) is accepted.

## Blinding

- Blinding of participants and investigators to treatment allocation is not possible due to the study setup.
- All baseline assessments will be conducted before randomization.
- Reading of the primary outcome CFVR and TTSDE at follow-up will be blinded.
- VO_2_max test at follow-up will be blinded to the conducting physiotherapist.
- DEXA scan at follow-up will be blinded to the conducting MD.

## Intervention

The components and goals of the intervention are outlined in **Table 2.** The main

components are:

- Body weight loss without significant loss of muscle mass, through low energy diet combined with
- Exercise training based on interval training and resistance exercise^20^
- Optimal medical treatment for hypertension and hypercholesterolemia (with statin and ACE-inhibition)
- Risk factor control: smoking cessation aid, nutrition counselling, screening and treatment for diabetes

DEXA scan and exercise capacity measurements are primarily used to ensure and monitor efficiency of exercise and weight loss interventions. The intervention program involves a multidisciplinary approach provided by a team of dieticians, physiotherapists and doctors trained in cardiac rehabilitation. Attention will be given to program flexibility combining structured group-based protocols with individually tailored strategies.

## Table 2. Overview of the components of the 24-week intervention

|  | Target group | Duration | Intervention | sessions | Goal | Responsible |
| --- | --- | --- | --- | --- | --- | --- |
| **Exercise training**  **Week 0-24** | **All** | **1½ h** | **Twice a week** | **Group** | **VO_2_ peak 10% improved** | **Card rehab team: Physiotherapist** |
| **Weight loss**  **Week 0-12** | **All** | **1 h** | **Every second week**  **+**  **1 individual** | **Group**  **+**  **individual** | **10% weight loss** | **Card rehab team: Dietician** |
| **Weight maintenance**  **Week 12-24** | **All** | **1 h** | **Monthly**  **+**  **1 individual** | **Group**  **+**  **individual** | **Heart healthy diet** | **Card rehab team: Dietician** |
| **Smoking cessation** | **Smokers**  **33%** |  | **Pharmacological support** | **Group** | **Quit smoking** | **MUNICIPAL** |
| **Optimized medication** | **LDL > 2**  **SBT >130**  **HbA1c >6.0^*^** | **½ h** | **Statin**  **ACE blocker**  **Metformin** | **Individual** | **LDL < 2**  **BP 130/80**  **HbA1c < 6.5** | **Clinical assistant, MD** |

^*^ Newly diagnosed DM2

##

## Figure 4. Participants’ study plan

## Figure 5. Antihypertensive treatment algorithm

### Exercise training (ET)

ET consists of group sessions of aerobic interval training and resistance exercise, scheduled twice weekly throughout the 24-week intervention period. Each group consists of 10 participants and each session lasts for 1 h. The group sessions are supervised and monitored by a physiotherapist experienced in cardiac rehabilitation. The intensity of the training will be individually adapted to the concomitant weight loss program with restricted calorie intake. During the first 12 weeks of calorie restriction, the training will be modest in load and intensity and primarily consist of short aerobic interval sessions and resistance exercise of the large muscle groups.

Attendance at training sessions will be registered by the physiotherapist. Home based training is accepted, if participants cannot attend all the sessions. Compliance of home-based training is self-reported (registered by a telephone call to the participant). Lack of compliance is defined as < 50% attendance to training sessions/home training. Lack of compliance within the first five weeks of intervention will lead to withdrawal from the study and possibly new inclusion.

**Please see Figure 6** for an overview of the estimated timeline of the training sessions throughout the study period. The 30 participants in the intervention group attend at 3 teams of 10 participants.

## Figure 6. Overview of exercise training group sessions


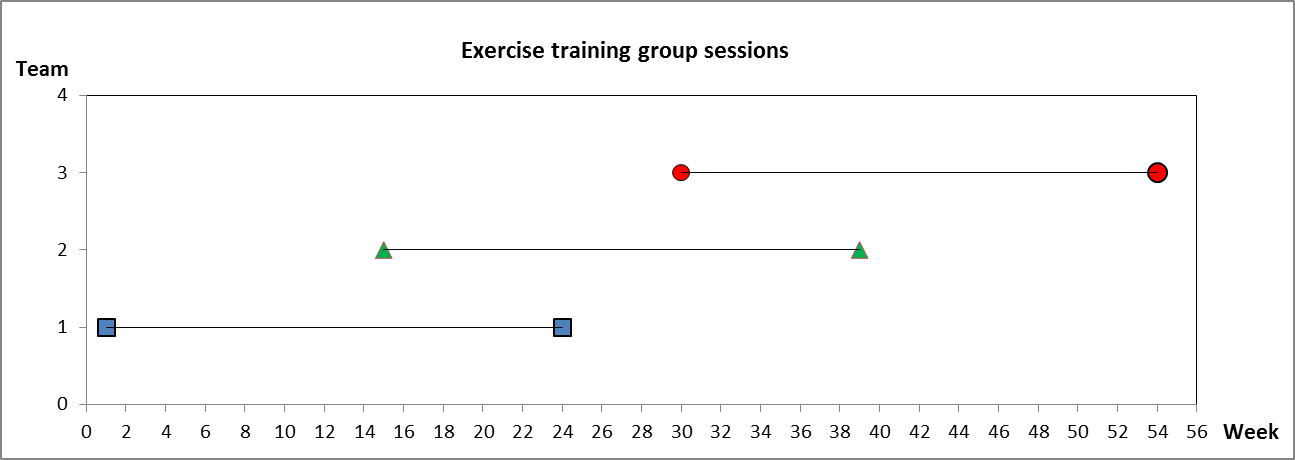


### Low energy diet (LED)

Weight loss will be achieved by following an evidence-based LED formula delivered by Cambridge Weight Plan. The diet, consisting of shakes, soups and bars, allows the participant to consume 800-1200 kcal/day. The diet contains the daily need of energy as well as macro- and micronutrients, proteins, essential fatty acids and recommended daily doses of vitamins and minerals^29^. The goal of the intervention is a total weight loss of at least 10% without significant loss of muscle mass.

Participants will be offered comprehensive support to fulfill the weight loss through a combination of two individual meetings and group sessions with a dietician. Attendance in a group session (5-7 participants and a dietician) defines the beginning of the 24-week interventional period for each participant. The purpose of this meeting is information about the LED treatment. Hereafter, participants are offered a single individual meeting with the dietician in order to register weight and dispense the Cambridge Weight Plan diet. Participants then follow a 12-week period with LED and attend group sessions every second week with their team and a dietician. The purpose of the group sessions is to motivate, adjust treatment to individual concerns, and monitor weight and compliance to intervention. During the 12^th^ week, participants will be offered another individual meeting with the dietician in order to motivate and advise in the weight maintenance diet.

The recommended weight maintenance diet is high in protein and with a low glycemic index modified from the DiOGenes study.^30^ Taking into consideration that the participants have CMD the high protein diet is adapted to resemble the Mediterranean diet, which is low in saturated fat (< 10%) and recommended to heart patients,^31^ with protein sources mainly from fish, poultry, egg, dairy, and vegetables. During the 12 weeks of weight maintenance the participants will be encouraged to still attend the monthly group sessions with the dietician. In case of weight gain LED can be used to replace meals. No weight loss within the first three weeks of LED is considered as lack of compliance and leads to withdrawal from the study.

The dietary group sessions will be scheduled at baseline (week 0) and week 1, 3, 5, 7, 9, and 11 and last for 60 minutes. In the weight maintenance period from week 12-24 the sessions will be scheduled monthly. The two individual sessions with dietician will scheduled in week 0-2 and optimally in week 12 and last for 60 minutes. Please see **Figure 7** for a schematic overview.

## Figure 7. Overview of diet sessions


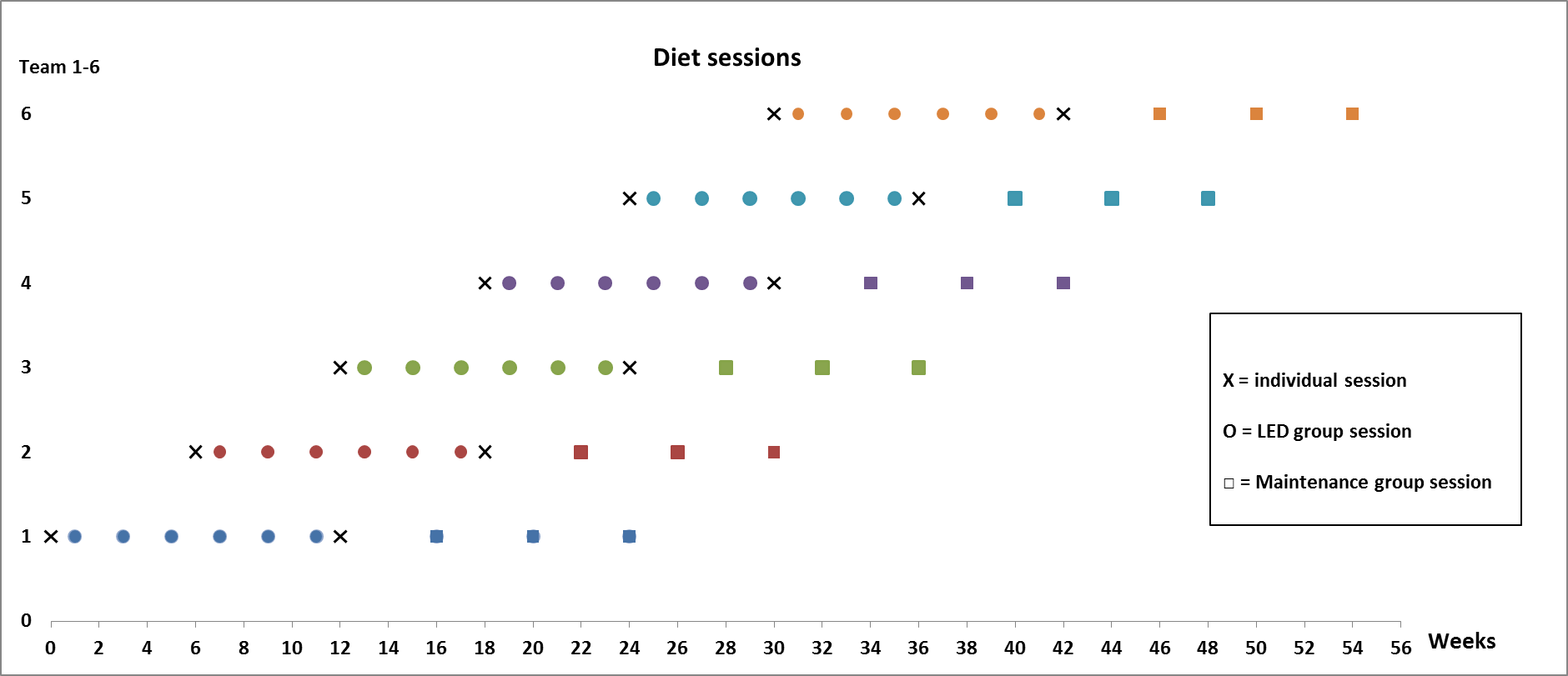


Both ET and dietician sessions will be conducted at Frederiksberg Hospital, Dept. of Cardiology.

### Controls – usual care

Usual care for patients with no CAD disease is normally provided by the patient’s general practitioner and does not comprise intensive lifestyle intervention or medical treatment. If controls in this study need medical therapy for hypertension or hypercholesterolemia, this will be effectuated by the researcher, MD. Then, controls will be monitored with blood pressure and LDL blood samples and receive medicine adjustments according to National Prevention Guidelines (NBV) during the full study period.

## Description of procedures

### Coronary flow velocity reserve (CFVR), systolic and diastolic function:

Coronary flow reserve is a measure of microvascular dysfunction in the absence of upstream coronary stenosis. CFVR is the ratio of flow during stress and flow during rest and will be measured with transthoracic Doppler stress echocardiography (TTDSE) of the left anterior descending artery (LAD) before and during infusion of high dose dipyridamole (0.84 mg/kg) or adenosine.

The feasibility of CFVR measurement by TTDSE has been tested on the iPower population (angina and no macrovascular coronary stenosis) and could be reliably assessed on the LAD with good quality in 97% of the population.^32^ CFVR measured by this method has been shown to be a strong risk marker in patients with non-obstructive coronary artery disease.^14^ In a study of 394 patients with chest pain and no angiographic stenosis, CFVR below 2 assessed by Doppler was associated with a hazard ratio of 16 for subsequent death or nonfatal myocardial infarction.^13^ The TTDSE method has shown to be highly reliable. In our previous validation study, 10 young healthy subjects were examined by the same observer with repeated TTDE CFVR. We found an intra class correlation coefficient of 0.97 (95% CI 0.92- 1.00) and coefficient of variation of 7% (95% CI 3-10%) for repeat examinations. CFVR reading for the two observers were highly reproducible in a subsample of 50 patients from the iPower cohort.^28^ A validation of the method in an overweight population comprised partly of the CUT-IT population (Coronary artery disease and non-diabetics) and patients awaiting cardiac rehabilitation. Here, the method showed high feasibility with successful measurements in 97% of 86 participants. The reproducibility was acceptable with reliability of 0.84, RC of 0.70 and within subject coefficient of variation of 11% with better reproducibility for exams repeated within a week (Olsen RH et al, unpublished data).

### **Echocardiography – strain**

Diastolic heart function tends to be impaired in obesity and in diabetes^33^ but whether this is also the case in microvessel dysfunction is unclear.

Improvement in cardiac function following intervention may be subtle and is more likely measurable during stress. By using global longitudinal and radial 2D strain at rest and during dipyridamole or adenosine stress, we expect that we will be able to detect a smaller difference in myocardial function than by using change in LVEF assessed by the Simpsons method.

### Exercise capacity (VO_2_ peak)

A cardiopulmonary exercise test (CPET) is performed using a bicycle ergometer (Corival, Lode, Germany) with breath-by-breath gas exchange measurements (Jaeger, Vyntus CPX, Germany. Participants will be encouraged to continue until exhaustion. Criteria for VO_2_peak are levelling off of VO_2_ despite increasing workload and peak respiratory exchange ratio (peak RER) > 1.10.^38^ VO_2_peak and peak RER are determined at peak effort with 15-second average measurements. ^39^ VO_2_peak is expressed as: VO_2_peak (mL/min), VO_2_peak (mL/kg body weight/min) and VCO_2_peak (mL/min).^40^ Predicted VO_2_peak is calculated using the equation for sedentary, overweight individuals presented by Wassermann and Hansen.^39^ Measured VO_2_peak in percent of predicted is determined.

### Dexa scan and body composition

The loss of lean body mass should be considered when planning a weight loss protocol since a decrease in lean body mass has been linked to increased mortality.^41^ ^42^ To estimate body composition (body fat mass and fat free mass ) a whole body dual X-ray absorptiometry (DEXA) scan will be performed.

Body composition, body weight, hip and waist circumference will be measured in the morning after a 10-hour fast. Waist circumference is measured halfway between the lower rib and the iliac crest and hip circumference at the maximal gluteal protuberance and calculated as an average of two consecutive measurements.

### Clinical examination

Blood pressure, heart rate, weight.

### Questionnaires:

In research settings the Seattle Angina Questionnaire and Hospital Anxiety and Depression Scale are the most commonly used, validated questionnaires for assessing angina symptom burden and anxiety and depression, respectively.

### Seattle Angina Questionnaire - SAQ

Improving quality of life is one of the primary goals in treating patients with stable angina pectoris. Quality of life is increasingly used as a patient-centered endpoint in cardiovascular disease studies, especially for stable ischemic heart disease. From a patient point of view angina may be the most important factor reducing quality of life, and data suggest that angina frequency predicts higher rates of cardiovascular events and death.^43^ Angina symptoms may not always be associated with an impaired CFVR, and conversely^44^ but interventions with ACE-I^18^ or weight loss ^45^ have improved angina symptoms (assessed by SAQ or Canadian Cardiovascular Society, CSS) and CFVR in previous studies.

The SAQ is a reliable, predictive tool that has been validated in 175 women with a confirmed diagnosis of stable coronary artery disease (CAD) and angina pectoris ^46^. It is a 19-item health-related quality-of-life measure for patients with coronary artery disease. The answers given by the patients in the SAQ’s questions are used to calculate scores in five scales:

- Anginal Stability: Whether a patient’s symptoms are changing over time.
- Anginal Frequency: Present frequency of symptoms.
- Physical Limitation: How much a patient’s condition is hampering her physical abilities.
- Treatment Satisfaction: How well a patient understands and what she thinks of it.
- Quality of Life: A measure of the overall impact of a patient’s condition on her interpersonal relationships and state of mind.

Scale scores are transformed to a 0-100 range by subtracting the lowest possible scale score, dividing by the range of the scale and multiplying by 100. Higher scores indicate less symptom burden. Because each scale monitors a unique dimension of coronary artery disease, no summary score is generated. A score change of 10 points is clinically perceptible to patients and is considered a clinically relevant difference, while a substantial change is considered to be a change of 20 points^47^ (please see Appendix for SAQ).

### Hospital Anxiety and Depression Scale – HADS

HADS is a self-administered questionnaire consisting of 14 items (each scored 0–3), seven of which concern depression and seven anxiety symptoms.^48^ One of the main purposes of this instrument was to identify affective symptoms among somatically ill patients.^49^ Therefore, the items focuses on the non-somatic aspects of depression and anxiety, to avoid that symptoms from the somatic disease, such as fatigue, affected the measurements.^48^ ^50^ Depression and anxiety items are summarized separately in two scales ranging from 0 to 21, where a higher score indicates more symptoms.

### Biomarkers

Several components in the inflammatory system may be associated with cardiovascular disease and atherosclerosis. A meta-analysis comprising 29 prospective studies associated interleukin-6 (IL6), tumor necrosis factor-alfa (TNF-alfa) and C-reactive protein (CRP) to increased cardiovascular risk independent of traditional risk factors in the healthy population.^51^

Fasting blood samples will be collected at baseline and follow-up for assessment of:

- Lipids (total-, low density lipoprotein, very low density lipoprotein, high density lipoprotein, cholesterol and triglycerides)
- Endocrine function (HbA1c, glucose, insulin, thyroid stimulating hormone)
- Kidney function (creatinine, eGFR)
- Haematology
- Inflammatory disease (hsCRP, hsTNT, orosomucoid, IL-6, TNF-alfa, adiponectine) biomarkers for later analysis

## Biological material

Two types of biobanks will be established

1. Research biobank for analysis of the material within the framework of the study.
2. Biobank for future research. The material can only be used with a renewed approval from the patients.

For every patient, 20 ml of the drawn 50 ml blood is stored in a biobank for biomarker analysis. Blood samples will be stored until the end of the study (last patient, last visit) plus five years and they will be destroyed thereafter. This will be registered in the Danish Data Protection Agency.

Serum, plasma and full blood will be stored in a -80^o^C fridge with an alarm mechanism that goes off in case the temperature rises above -60^o^C. The cryotubes are kept in 9x9 consecutively numbered boxes, separated as Serum, Plasma and Full blood. The content of each box is noted in an electronic file, coupling patient ID, box number and number of cryotubes.

# Statistics

## Sample size

The primary endpoint is change in microvascular function assessed by CFVR.

We wish to be able to detect a difference in change in CFVR between groups of 10%, equal to a change in CFVR of 0.24, assuming a baseline mean CFVR of 2.4. According to our previous study (22) the standard deviation of the within individual change is assumed to be 0.3. With power of 0.8 and two-sided significance level of 0.05, 26 patients should be included in each group. With 26 in each patient group we will be able to detect a moderate or larger effect on symptom burden. Anticipating dropout, randomization of a total of 60 patients was chosen. Dropout within the first three weeks of inclusion is replaced by new inclusion.

SAQ has four subscales each with a score from 1-100. We have not found data on within-study subject variation on SAQ. However, previous trials have obtained changes in SAQ of 10-20 points after intervention.^18^ ^46^ ^13^ A trial of 46 patients (predominantly women) with microvascular angina randomized to either ranolazine, ivabradine or placebo for four weeks found a SAQ score change of 10-40 points across different SAQ parameters, all highly significant (p<0.001).^18^ We will conduct a study on a similar study population, and we therefore expect to have the statistical power to document clinically relevant effects.

With 60 participants included, the probability is 80 percent that the study will detect a treatment difference at a two-sided 0.05 significance level, if the true difference between treatments is 11. This is based on the assumption that the standard deviation of the response variable is 15.

Analyses will be performed in STATA/IC 13.1. Categorical data will be presented as number (percentage), continuous data as mean (SD) or median (interquartile range) if normal distribution cannot be assumed. Regression analysis will be performed with the primary outcome change in symptoms (SAQ) and the independent variable change in CFVR.

# Risks and disadvantage for participants

According to previous trials, patients with microvessel disease have an increased risk of cardiovascular mortality and morbidity. To date no approved treatment for microvessel disease exists. This study may add to the understanding of microvessel disease and possible treatment options with a direct effect on the microvasculature. By improving microvessel function the progression of cardiovascular disease may be prevented in these patients. At the individual level, participants randomized to intervention in this study will hopefully benefit from the comprehensive support and treatment team towards a more heart-healthy lifestyle and increased quality of life. By this intervention it is possible for participants to improve their physical condition, achieve fat reduction, especially the visceral fat surrounding the heart and other vital organs and thereby reduce risk factors for cardiac disease, among these hypertension, diabetes and metabolic syndrome. Risks and disadvantages are considered offset by the potential benefits of the study.

Expected disadvantages are discomfort/pain in relation to blood testing, risk of following rash or bruises that are considered harmless and will disappear after a few days.

Adenosine, which might be used during the CFVR measurements instead of dipyridamole, has known side effects similar to dipyridamole. Adenosine side effects are observed more frequently, however, they normally last for only one minute. In case of severe disadvantage for the participant in relation to infusion of adenosine an antidote, Teofyllamine, will be given. Hereafter symptoms usually diminish within minutes.

Exercise training will be monitored by a physiotherapist with experience in cardiac rehabilitation. There will be supportive health educated staff in the building in case of emergency as well as resuscitation equipment. Participating in exercise training is considered safe.

The low calorie diet with Cambridge Weight Plan has been used in studies of overweight patients without being associated with health risk.^52^ The diet intervention will be guided by a clinical dietician experienced in cardiac rehabilitation.

By Dexa scan the participant is exposed to a radiation dose of about 0.005 mSv which is considered insignificant. In comparison the annual background radiation in Denmark is

2 - 20 mSv depending on location.

# Ethical aspects

## Regulatory authorities

The trial will be registered at clinicaltrials.gov before inclusion begins and requires approval by the local regulatory authorities The National Committee on Health Research Ethics and The Danish Data Protection Agency. These authorities will be given direct access to data by the investigator. The study adheres to the Helsinki Declaration.

## Informed consent

Patients are selected from the iPower cohort on the basis of in- and exclusion criteria. Only patients who fulfil criteria will be contacted and those who have given permission to contact at inclusion in iPower. Written information about the project will be sent by post or delivered by hand to the patients (Appendix: “Rekrutteringsmateriale”), and afterwards they will be contacted by phone. They will be informed orally about the project and if they are interested, an appointment will be arranged, and a second letter will be send with additional information, questionnaires and confirmation of the appointment date (Appendices “Deltagerinformation” and “Deltagerbrev 1”).

Patients will be given a few days between oral information and signing the informed consent to consider whether they wish to participate in the study. Participants will also be informed both orally and written about the possibility of an accompanying relative at the day of appointment. On the day of appointment study information will be given in a quiet room with only the primary researcher, MD, the participant and eventually her accompanying relative(s) present. Informed consent document will be signed and collected at this meeting.

Information about study participants will be protected in accordance with the Danish Data Protection Agency. Participants will be assured that participation is voluntary and that withdrawal without explanation is fully accepted at any point of the study if participants do not wish to continue.

## Data storage

Every patient will receive an individual study number. All data on every patient will be registered under this number. All data will be stored securely in a locked office and in an electronic database for five years.

## Organization of the study

This research project is an extension of the ongoing iPower study, which is a Danish

multicentre study supervised by a steering committee with representatives from each of the

five collaborating Danish centres. The trial will be conducted at The Cardiac

Rehabilitation unit at the Department of Cardiology, Bispebjerg/Frederiksberg hospital

(www.hjerterehabilitering.dk), which has the largest patient uptake in Denmark and has

extensive experience with conducting intervention trials in cardiac rehabilitation. A web-based electronic case record program has been developed in accordance with Danish

data legislation by TrialPartner, Centre for Public Health, Central Denmark Region, which

will also be responsible for randomization, which is thus independent in accordance with

good clinical practice.

# Costs

## Table 3. Overview of estimated costs of the 24-week intervention

| **24-week intervention** | **Item in intervention** | **Time (hours)** | **Salary/hour** | **Costs (DKK)** |
| --- | --- | --- | --- | --- |
| ET group sessions (physio) | 3h/week x 24 weeks x 3 teams of 8 participants | 216 | 200 | 43.200 |
| VO_2_max test (CPET) baseline + follow-up (physio) | 45 min/participant x 130* | 97½ | 200 | 19.500 |
| LED group sessions (dietician) | 1 h x 6 teams x 9 times | 54 | 254 | 13.716 |
| Weight registration e.g.  (dietician) | ½ h x 54 times | 27 | 254 | 6.858 |
| Weight loss individual sessions (dietician) | 1 h/participant x 70* times | 70 | 254 | 17.780 |
| Cambridge Weight Plan Diet | 3 meals daily x 12 weeks x 70 participants* | 0 | Sponsored | 0 |
| Smoking cessation group sessions (nurse) | 3 sessions x 1 h  (10 participants) | 0 | Municipal | 0 |
| **Total costs, all patients** |  |  |  | **101.054** |

*10 participants extra in case of drop-out and new inclusion. CPET includes control group participants.*

## Table 4. Total costs of the research project

| **Item** | **Financed by BBH** | **Financed by other fonds** | **Needs to be applied for** | **Total costs (DKK)** |
| --- | --- | --- | --- | --- |
| Interventional program  (see table 4) |  | 100.000 |  | 100.000 |
| Cambridge Weight Plan Diet |  | Sponsored |  | 0 |
| PhD salary, 3 years  (see table 5) |  | 660.000 | 912.014 | 1.572.014 |
| KU PhD tuition fee  3 years x 60.000 DKK |  | 180.000 |  | 180.000 |
| Analysis of biomarkers  2 x 60 x 500 DKK |  |  | 60.000 | 60.000 |
| Blood samples  200 x 500 DKK | 100.000 |  |  | 100.000 |
| Medical treatment  (see table 3) | 3.000 |  |  | 3.000 |
| PC and programs  (Stata, Office)  10.000 + 15.000 + 1.000 | 26.000 |  |  | 26.000 |
| Research assistant  12 x 10.000 DKK |  | 120.000 |  | 120.000 |
| **Total costs** | 129.000 | 40.000 (CCF) + 900.000 (FUNC)+ 100.000 (BBH)+ 20.000 (iPOWER) | 912.014 | 2.161.014 |

The National Committee on Health Research Ethics and study participants will be informed if further funding is achieved.

# Publication of results

Both positive, negative and inconclusive data from this study will be published in indexed, , medical journals with peer review and as abstracts at congresses.

# References

1. Bairey Merz CN, Shaw LJ, Reis SE, et al. Insights From the NHLBI-Sponsored Women’s Ischemia Syndrome Evaluation (WISE) Study. *J Am Coll Cardiol*. 2006;47(3):S21-S29. doi:10.1016/j.jacc.2004.12.084.

2. Shaw LJ, Bugiardini R, Merz CNB. Women and ischemic heart disease: evolving knowledge. *J Am Coll Cardiol*. 2009;54(17):1561-1575. doi:10.1016/j.jacc.2009.04.098.

3. Patel MR, Peterson ED, Dai D, et al. Low diagnostic yield of elective coronary angiography. *N Engl J Med*. 2010;362(10):886-895. doi:10.1056/NEJMoa0907272.

4. Jespersen L, Hvelplund A, Abildstrøm SZ, et al. Stable angina pectoris with no obstructive coronary artery disease is associated with increased risks of major adverse cardiovascular events. *Eur Heart J*. 2012;33(6):734-744. doi:10.1093/eurheartj/ehr331.

5. Gulati M, Cooper-DeHoff RM, McClure C, et al. Adverse Cardiovascular Outcomes in Women With Nonobstructive Coronary Artery Disease. *Arch Intern Med*. 2009;169(9):843-850. doi:10.1001/archinternmed.2009.50.

6. Jespersen L, Abildstrøm SZ, Hvelplund A, et al. Symptoms of angina pectoris increase the probability of disability pension and premature exit from the workforce even in the absence of obstructive coronary artery disease. *Eur Heart J*. 2013;34(42):3294-3303. doi:10.1093/eurheartj/eht395.

7. Jespersen L, Abildstrøm SZ, Hvelplund A, Prescott E. Persistent angina: highly prevalent and associated with long-term anxiety, depression, low physical functioning, and quality of life in stable angina pectoris. *Clin Res Cardiol*. 2013;102(8):571-581. doi:10.1007/s00392-013-0568-z.

8. Shaw LJ, Merz CNB, Pepine CJ, et al. The economic burden of angina in women with suspected ischemic heart disease: results from the National Institutes of Health--National Heart, Lung, and Blood Institute--sponsored Women’s Ischemia Syndrome Evaluation. *Circulation*. 2006;114(9):894-904. doi:10.1161/CIRCULATIONAHA.105.609990.

9. Crea F. Angina pectoris and normal coronary arteries: cardiac syndrome X. *Heart*. 2004;90(4):457-463. doi:10.1136/hrt.2003.020594.

10. Bugiardini R et al. Angina With “ Normal ” Coronary Arteries. *JAMA*. 2005;293(4):477-484.

11. Phan A et al. Persistent Chest Pain and No Obstructive Coronary Artery Disease. *JAMA*. 2009;14(14):1468-1474.

12. Cannon RO. Microvascular Angina and the Continuing Dilemma of Chest Pain With Normal Coronary Angiograms. *J Am Coll Cardiol*. 2009;54(10):877-885. doi:10.1016/j.jacc.2009.03.080.

13. Sicari R, Rigo F, Cortigiani L, Gherardi S, Galderisi M, Picano E. Additive Prognostic Value of Coronary Flow Reserve in Patients With Chest Pain Syndrome and Normal or Near-Normal Coronary Arteries. *Am J Cardiol*. 2009;103(5):626-631. doi:10.1016/j.amjcard.2008.10.033.

14. Pepine CJ, Anderson RD, Sharaf BL, et al. Coronary Microvascular Reactivity to Adenosine Predicts Adverse Outcome in Women Evaluated for Suspected Ischemia. *J Am Coll Cardiol*. 2010;55(25):2825-2832. doi:10.1016/j.jacc.2010.01.054.

15. Rigo F, Cortigiani L, Pasanisi E, et al. The additional prognostic value of coronary flow reserve on left anterior descending artery in patients with negative stress echo by wall motion criteria. A Transthoracic Vasodilator Stress Echocardiography Study. *Am Heart J*. 2006;151(1):124-130. doi:10.1016/j.ahj.2005.03.008.

16. Ong P, Athanasiadis A, Borgulya G, Mahrholdt H, Kaski JC, Sechtem U. High prevalence of a pathological response to acetylcholine testing in patients with stable angina pectoris and unobstructed coronary arteries: The ACOVA study (abnormal coronary vasomotion in patients with stable angina and unobstructed coronary arteries. *J Am Coll Cardiol*. 2012;59(7):655-662. doi:10.1016/j.jacc.2011.11.015.

17. Murthy VL, Naya M, Foster CR, et al. Improved Cardiac Risk Assessment With Noninvasive Measures of Coronary Flow Reserve / Clinical Perspective. *Circulation*. 2011;124(20):2215-2224. doi:10.1161/circulationaha.111.050427.

18. Pauly DF, Johnson BD, Anderson RD, et al. In women with symptoms of cardiac ischemia, nonobstructive coronary arteries, and microvascular dysfunction, angiotensin-converting enzyme inhibition is associated with improved microvascular function: A double-blind randomized study from the National Hea. *Am Heart J*. 2011;162(4):678-684. doi:10.1016/j.ahj.2011.07.011.

19. Villano A, Di Franco A, Nerla R, et al. Effects of Ivabradine and Ranolazine in Patients With Microvascular Angina Pectoris. *Am J Cardiol*. 2013;112(1):8-13. doi:10.1016/j.amjcard.2013.02.045.

20. Pedersen LR, Olsen RH, Frederiksen M, et al. Copenhagen study of overweight patients with coronary artery disease undergoing low energy diet or interval training: the randomized CUT-IT trial protocol. *BMC Cardiovasc Disord*. 2013;13:106. doi:10.1186/1471-2261-13-106.

21. Olsen RH et al. Europevent EACPR annual congress 2014. *Eur EACPR Annu Congr*. 2014.

22. Hambrecht R et al. Effect of exercise on coronary endothelial function in patients with coronary artery disease. *N Engl J Med*. 2000;342(7):454-460.

23. Hambrecht R, Walther C, M??bius-Winkler S, et al. Percutaneous Coronary Angioplasty Compared with Exercise Training in Patients with Stable Coronary Artery Disease: A Randomized Trial. *Circulation*. 2004;109(11):1371-1378. doi:10.1161/01.CIR.0000121360.31954.1F.

24. Reriani MK, Dunlay SM, Gupta B, et al. Effects of statins on coronary and peripheral endothelial function in humans: a systematic review and meta-analysis of randomized controlled trials. *Eur J Cardiovasc Prev Rehabil*. 2011;18(5):704-716. doi:http://dx.doi.org/10.1177/1741826711398430.

25. Kerr SM, Livingstone MB, McCrorie TA WJ. Endothelial dysfunction associated with obesity and the effect of weight loss interventions. *Proc Nutr Soc*. 2011;70(4):418-425.

26. Diabetes Prevention Program Research Group. 10-year follow-up of diabetes incidence and weight loss in the Diabetes Prevention Program Outcomes Study. *Lancet*. 2009;374(9702):1677-1686. doi:10.1016/S0140-6736(09)61457-4.

27. Galderisi M, D’Errico A. Beta-blockers and coronary flow reserve: the importance of a vasodilatory action. *Drugs*. 2008;68(5):579-590. doi:6852 [pii].

28. Prescott E, Abildstrøm SZ, Aziz A, et al. Improving diagnosis and treatment of women with angina pectoris and microvascular disease: The iPOWER study design and rationale. *Am Heart J*. 2014;167(4):452-458. doi:10.1016/j.ahj.2014.01.003.

29. Leeds AR. Formula food-reducing diets: A new evidence-based addition to the weight management tool box. *Nutr Bull*. 2014;39(3):238-246. doi:10.1111/nbu.12098.

30. Larsen TM, Dalskov S-M, van Baak M, et al. Diets with high or low protein content and glycemic index for weight-loss maintenance. *N Engl J Med*. 2010;363(22):2102-2113. doi:10.1056/NEJMoa1007137.

31. Ramon Estruch, M.D., Ph.D., Emilio Ros, M.D., Ph.D.Jordi Salas-Salvado, M.D. PD, Maria-Isabel Covas, D.Pharm., Ph.D., Dolores Coreila, D.Pharm., Ph.D., Fernando Ards, M.D., Ph.D., Enrique G6mez-Gracia, M.D., Ph.D., Valentina Ruiz-Gutierrez, Ph.D., Miquel Fiol, M.D. PD, Jos^ Lapetra, M.D., Ph.D., Rosa Maria Lamuela-Raventos, D.Pharm., Ph.D., LIuis Serra-Majem, M.D., Ph.D., Xavler Pinto, M.D., Ph.D., Josep Basora, M.D., Ph.D., Miguel Angel Munoz, M.D., Ph.D., Jos6 V. Sorli, M.D. PD, Jos^ Alfredo Martinez, D.Pharm, M.D., Ph.D., and Miguel Angel Martinez-Gonzalez, M.D., Ph.D. for the PSI-. Primary Prevention of Cardiovascular Disease with a Mediterranean Diet. *N Engl J Med*. 2013;368(14):1279. doi:10.1056/NEJMoa1200303.

32. Mygind ND/Michelsen MM, Pena A, Frestad D, Dose N, Aziz A, Faber R, Høst N, Gustafsson I, Hansen PR, Hansen HS, Bairey Merz N, Kastrup J PE. Coronary microvascular function and cardiovascular risk factors in women with angina pectoris and no obstructive coronary artery disease:the iPOWER study. *Accept JAHA*. 2016.

33. Banerjee S, Peterson LR. Myocardial Metabolism and Cardiac Performance in Obesity and Insulin Resistance Corresponding author. *Obes Surg*. 2007;2000.

34. Vita JA. Endothelial Function. *Circulation*. 2011;124(25):e906-e912. doi:10.1161/CIRCULATIONAHA.111.078824.

35. Anderson TJ, Uehata A, Gerhard MD, et al. Close relation of endothelial function in the human coronary and peripheral circulations. *J Am Coll Cardiol*. 1995;26(5):1235-1241. doi:10.1016/0735-1097(95)00327-4.

36. Corretti MC, Anderson TJ, Benjamin EJ, et al. Guidelines for the ultrasound assessment of endothelial-dependent flow-mediated vasodilation of the brachial artery: a report of the International Brachial Artery Reactivity Task Force. *J Am Coll Cardiol*. 2002;39(2):257-265. doi:10.1016/S0735-1097(01)01746-6.

37. Sasaki S, Higashi Y, Nakagawa K, et al. A low-calorie diet improves endothelium-dependent vasodilation in obese patients with essential hypertension. *Am J Hypertens*. 2002;15(4 I):302-309. doi:10.1016/S0895-7061(01)02322-6.

38. Guazzi M et al. CardioPulse. New clinical cardiopulmonary exercise testing joint statement from the European Society of Cardiology and American Heart Association. *Eur Hear J*. 2012;33(21):2627-2628. doi:10.1093/eurheartj/ehs319 ehs319 [pii] ET - 2012/11/03.

39. Guazzi M, Adams V, Conraads V, et al. Clinical recommendations for cardiopulmonary exercise testing data assessment in specific patient populations. *Eur Heart J*. 2012;33(23):2917-2927. doi:10.1093/eurheartj/ehs221.

40. von Döbeln W. Maximal oxygen intake, body size, and total hemoglobin in normal man. *Acta Physiol Scand*. 1956;38(2):193-199. doi:10.1111/j.1748-1716.1957.tb01383.x.

41. Schutter A De, Lavie CJ, Kachur S, Patel DA, Milani R V. Body composition and mortality in a large cohort with preserved ejection fraction: Untangling the obesity paradox. *Mayo Clin Proc*. 2014;89(8):1072-1079. doi:10.1016/j.mayocp.2014.04.025.

42. Lavie CJ, De Schutter A, Patel DA, Romero-Corral A, Artham SM, Milani R V. Body composition and survival in stable coronary heart disease: Impact of lean mass index and body fat in the “obesity paradox.” *J Am Coll Cardiol*. 2012;60(15):1374-1380. doi:10.1016/j.jacc.2012.05.037.

43. Beatty AL, Spertus J a., Whooley M a. Frequency of Angina Pectoris and Secondary Events in Patients With Stable Coronary Heart Disease (from the Heart and Soul Study). *Am J Cardiol*. 2014;114(7):997-1002. doi:10.1016/j.amjcard.2014.07.009.

44. Di Franco A, Villano A, Di Monaco A, et al. Correlation between coronary microvascular function and angina status in patients with stable microvascular angina. *Eur Rev Med Pharmacol Sci*. 2014;18(3):374-379.

45. Pedersen LR, Olsen RH, Jürs A, et al. A randomised trial comparing weight loss with aerobic exercise in overweight individuals with coronary artery disease: The CUT-IT trial. *Eur J Prev Cardiol*. 2014:DOI:10.1177/2047487314545280. doi:10.1177/2047487314545280.

46. Kimble LP, Dunbar SB, Weintraub WS, et al. The Seattle angina questionnaire: reliability and validity in women with chronic stable angina. *Heart Dis*. 2012;4(4):206-211. doi:10.1016/j.drudis.2011.09.009.

47. Spertus J a, Winder J a, Dewhurst T a, et al. Development and evaluation of the Seattle Angina Questionnaire: a new functional status measure for coronary artery disease. *J Am Coll Cardiol*. 1995;25(2):333-341. doi:10.1016/0735-1097(94)00397-9.

48. Snaith et al. The hospital anxiety and depression scale. *Acta Psychiatr*. 1983;67:361-370.

49. Herrmann C. International experiences with the Hospital Anxiety and Depression Scale--a review of validation data and clinical results. *J Psychosom Res*. 1997;42(1):17-41. doi:10.1016/S0022-3999(96)00216-4.

50. Snaith RP. Commentary. The Hospital Anxiety And Depression Scale. *Health Qual Life Outcomes*. 2003;1:29. doi:10.1186/1477-7525-1-29.

51. Kaptoge S, Seshasai SRK, Gao P, et al. Inflammatory cytokines and risk of coronary heart disease: New prospective study and updated meta-analysis. *Eur Heart J*. 2014;35(9):578-589. doi:10.1093/eurheartj/eht367.

52. Gilden Tsai A, Wadden TA. The Evolution of Very-Low-Calorie Diets: An Update and Meta-analysis. *Obesity*. 2006;14(8):1283-1293. http://www.obesityresearch.org/cgi/content/abstract/14/8/1283.

isord. 2013:13[1];106.

1. Definitions according to the guidelines of the Danish Society of Cardiology (DCS). [↑](#footnote-ref-1)
